# Profiling of MicroRNAs Involved in Retinal Degeneration Caused by Selective Müller Cell Ablation

**DOI:** 10.1371/journal.pone.0118949

**Published:** 2015-03-05

**Authors:** Sook Hyun Chung, Mark Gillies, Yuki Sugiyama, Ling Zhu, So-Ra Lee, Weiyong Shen

**Affiliations:** 1 Macular Research Group, Clinical Ophthalmology and Eye Health, Save Sight Institute, University of Sydney, Sydney, Australia; 2 Lens Research Group, Clinical Ophthalmology and Eye Health, Save Sight Institute, University of Sydney, Sydney, Australia; The University of Tennessee Health Science Center, UNITED STATES

## Abstract

Dysfunction of Müller cells has been implicated in the pathogenesis of several retinal diseases. In order to understand the potential contribution of Müller cells to retinal disease better, we have developed a transgenic model in which foci of Müller cell ablation can be selectively induced. MicroRNAs (miRNAs), small non-coding RNAs that are involved in post-transcriptional modulation, have critical functions in various biological processes. The aim of this study was to profile differential expression of miRNAs and to examine changes in their target genes 2 weeks after Müller cell ablation. We identified 20 miRNAs using the miScript HC PCR array. Data analysis using two target gene prediction databases (TargetScan and mirTarBase) revealed 78 overlapping target genes. DAVID and KEGG pathway analysis suggested that the target genes were generally involved in cell apoptosis, p53, neurotrophin, calcium, chemokine and Jak-STAT signalling pathways. Changes in seven target genes including Cyclin D2, Caspase 9, insulin-like growth factor 1, IL-1 receptor-associated kinase (IRAK), calmodulin (CALM) and Janus kinase 2 (Jak2), were validated with qRT-PCR and western blots. The cellular localisation of cleaved-caspase 9, Cyclin D2, Jak2 and CALM was examined by immunofluorescence studies. We found that the transcription of some miRNAs was positively, rather than negatively, correlated with their target genes. After confirming that overexpressed miR-133a-3p was localised to the outer nuclear layer in the damaged retina, we validated the correlation between miR-133a-3p and one of its predicted target genes, cyclin D2, with a luciferase assay in 661 photoreceptor cells. Results revealed by miRNA profiling, target gene analysis and validation were generally consistent with our previous findings that selective Müller cell ablation causes photoreceptor degeneration and neuroinflammation. Our data on alterations of miRNAs and their target gene expression after Müller cell ablation provide further insights into the potential role of Müller cell dysfunction in retinal disease.

## Introduction

Müller cells, the principal glial cells of the mammalian retina, are essential for retinal homeostasis. Spanning the entire thickness of the retina, they support the surrounding neurones, including photoreceptors [1, 2], and are involved in forming and maintaining the blood retinal barrier (BRB)[3]. Dysfunction of Müller cells has been implicated in many retinal diseases such as diabetic retinopathy and macular telangiectasia [4, 5]. To investigate the relationship between Müller cell dysfunction and retinal diseases, we have created a transgenic mouse model in which Müller cells can be selectively and inducibly ablated [6]. These mice develop photoreceptor degeneration, neuroinflammation, including activation of microglia and surviving Müller cells, and differential expression of mature and precursor forms of neurotrophic factors such as neurotrophin-3 within 2 weeks of Müller cell ablation [6, 7]. We have previously reported differential expression of genes that was consistent with many of these pathological processes in this model [2].

MicroRNAs (miRNAs) are small, non-coding RNA molecules which are ~22 nucleotide (nt) long which are in transcribed in the nucleus [8, 9]. They regulate post-transcriptional gene expression by binding to the complementary site of mRNA [10, 11]. The short hairpin structure of primary miRNAs are processed into pre-miRNA by RNase III-type enzyme, Drosha, in the nucleus and exported into the cytoplasm by exportin-5 [12]. Pre-miRNAs are catalyzed by Dicer, another kind of RNase III-type enzyme, in the cytoplasm to become ~22 nt mature miRNA [13]. Mature miRNAs are assembled into a ribonucleoprotein effector complex known as miRNA induced silencing complex and bind to proteins of the argonaute family and GW182 family [8, 10]. It was previously thought that the main function of miRNAs was to repress transcription and destabilize mRNA, however, recent studies have proposed that miRNAs can also stimulate target mRNA expression [14–17].

The role of miRNAs in diseases of the vertebrate retina is an area of considerable potential significance [18, 19]. Many studies have been focused on the pathophysiological role of miRNAs in the pathogenesis of retinal diseases such as diabetic retinopathy and retinitis pigmentosa [8, 20–23]. There are few studies, however, of differential expression of miRNAs in retinal diseases in which Müller cell dysfunction is suspected to play a role. The transgenic model described above, in which Müller cells can be selectively ablated, provides a unique opportunity to study alterations of retinal miRNAs after induced Müller cell ablation. The aims of this study were to profile the differential expression of miRNAs and their target genes, and to investigate changes in target gene expression 2 weeks after selective Müller cell ablation, which is the peak time of photoreceptor apoptosis in this model [6].

## Materials and Methods

### Animals

This study was performed in accordance with the Association for Research in Vision and Ophthalmology guidelines for animal research and approved by The University of Sydney Animal Ethics Committee. Rlbp1-CreER mice were crossed with Rosa-DTA176 mice to produce Rlbp-CreER-DTA176 transgenic mice. Müller cell ablation was induced by daily intraperitoneal injection of tamoxifen (TMX) for 4 consecutive days at 8–10 weeks of age [6]. Wild type mice receiving TMX in the same way were used as controls. A total of 12 mice, including 6 transgenic mice with induced Müller cell ablation and 6 wild type mice, were used in this study. Mice were euthanized 2 weeks after Müller cell ablation. Retinae were isolated, snap frozen in liquid nitrogen and stored at-80°C until use.

### miRNA Polymerase Chain Reaction (PCR) Array

Retinae were thawed then total RNA, including miRNAs, was extracted with a miRNeasy Mini Kit (Qiagen, 217004) according to manufacturer’s instructions. The quantity and quality were assessed by an Experion Automated Electrophoresis System (Biorad, 701–7001). Six retinae from each group were reverse-transcribed with miScript II RT kit (Qiagen, 218160) and diluted with RNase free water. miRNA PCR array was performed with 384 well-plate miScript miRNA High Content PCR array (Qiagen, MIMM-3001Z) which contains miScript primers for 372 of the most well characterised miRNAs and duplicates of 6 internal reference miRNAs. The identities of 372 miRNAs are listed in Supporting Information (Table A in S1 File). The thermo-cycle of the PCR array consisted of denaturing at 94° for 15 seconds, annealing at 55° for 20 seconds and extension at 70° for 30 seconds. Relative quantification was performed by the ΔΔC_T_ method recommended by the manufacturer (http://pcrdataanalysis.sabiosciences.com/mirna/arrayanalysis.php?target=analysis). A p-value <0.05 was considered statistically significant and the fold change cut-off criteria was ± 2.

### Functional Analysis of miRNAs

We started with functional analysis of each differentially expressed miRNAs by performing a PubMed literature search to identify recent publications, which would shed light on their related functions (http://www.ncbi.nlm.nih.gov/pubmed). In addition, we used 2 target gene prediction databases: TargetScan (http://www.targetscan.org) and miRTarbase (http://mirtarbase.mbc.nctu.edu.tw). Overlapping target genes from these 2 databases were identified for further analysis. Functional analysis of the target genes was performed by Database for Annotation, Visualisation Integrated Discovery (DAVID, http://david.abcc.ncifcrf.gov) and associated pathways were identified by KEGG pathway database (http://www.genome.jp/kegg/pathway.html). We also performed *in silico* study with mirTarBase to examine direct seed matching sequence alignments between the identified miRNAs and their target genes prior to further investigations (Table 1).

**Table 1:** Direct Sequence Alignment Between miRNAs and Their Target Genes 3’UTR.

ID	Target Gene	Binding Sites	Position	Target Score
miR133a-3p	Caspase 9	miRNA 3′ gucgACC—AACUU—-CCCCUGGUUu 5′	1706–1732	136.00
		||| || || | ||||||:		
		Target 5′ gtagTGGTCTTAAAAGTGTGGACCAGt 3′		
		miRNA 3′ gucGAC-CAACUUCC———-CCUGGUUu 5′	1416–1445	127.00
		: || ||| |: | ||:||||		
		Target 5′ gcaTTGAGTTCAGTGACTCTCAGGGCCAAg 3′		
		miRNA 3′ gucgACC-AACUUCCCCUGGUUu 5′	45–67	122.00
		||| |||:|| |:||||		
		Target 5′ ctcaTGGCTTGGAGCTGGCCAAg 3′		
miR133a-3p	Cyclin D2	miRNA 3′ guCGACCAACUUCCCCUGGUuu 5′	3974–3990	133.00
		||||| || ||||||		
		Target 5′ ggGCTGG—GA—-GGACCAca 3′		
		miRNA 3′ guCGACCAACUUCCCCUGGUUu 5′	3865–3883	125.00
		||| || ||||||| |		
		Target 5′ taGCTCTTT—-GGGGACCCAc 3′		
		miRNA 3′ gucGACCA—ACUUCCCCUGGuuu 5′	2963–2985	124.00
		||||| | || ||||||		
		Target 5′ cttCTGGTCCTTAA-GGGACCcca 3′		
miR133a-3p	Insulin like Growth Factor 1 Receptor	miRNA 3′ gucGACCAACUUC—CCCUGGUuu 5′	5357–5380	141.00
		|||| |||| ||||||		
		Target 5′ ggcCTGGAAGAAGCATGGACCAta 3′		
		miRNA 3′ gucgaccaacuuccCCUGGUUu 5′	5988–6009	140.00
		|||||||		
		Target 5′ agaaattctccccaGGACCAAt 3′		
		miRNA 3′ guCGAC-CAACUUCC——CCUGGUUu 5′	5022–5048	138.00
		|||| |||:: | ||||||:		
		Target 5′ caGCTGCATTGGGAGACCTGGACCAGa 3′		
miR-146a-5p	Interleukin Receptor Associated Kinase 1	miRNA 3′ uuGGGUACCUUAAG—UCAAGAGu 5′	29–50	154.00
		|:|| ||: || |||||||		
		Target 5′ gaCTCA—GAGGTCAAAGTTCTCa 3′		
		miRNA 3′ uuGGGUAC—-CUUAAGUCAAGAGU——— 5′	45–72	135.00
		|:|||| |:| ||||||||		
		Target 5′ ttCTCATGCTTGGA—-AGTTCTCATAGTGT 3′		
		miRNA 3′ uugGGUACCU—UAAGUCAAGAGu 5′	133–156	125.00
		|: |||| | | ||||||		
		Target 5′ gggCAGTGGACCCTGCTGTTCTCa 3′		
miR1a-1	Calmodulin	miRNA 3′ uaUGUAUGAAGAAAUGUAAGGu 5′	2743–2762	144.00
		||||| |:||||:||||:		
		Target 5′ gtACATA—TTTTTATATTCTc 3′		
		miRNA 3′ uaUG-UAUGAAG—AAAUGUAAGGu 5′	3350–3373	133.00
		||: || ||| | |||||:|		
		Target 5′ gcACTGTA-TTCAATAAACATTTCt 3′		
		miRNA 3′ uaUGUAUGAAG———AAAUGUAAGGu 5′	1234–1261	126.00
		||||: ||: | |::|||||		
		Target 5′ agACATGATTTAGTGTGTCTGTATTCCt 3′		
miR-375–3p	Janus Kinase 2	miRNA 3′ agugcgcUCGGCUUGCUUGUUu 5′	563–584	127.00
		||| || ||||||		
		Target 5′ ttttcaaAGCAAAAGGAACAAa 3′		
		miRNA 3′ agugcgcucggcuugCUUGUUu 5′	33–54	120.00
		||||||		
		Target 5′ agacttccagaaccaGAACAAa 3′		
		miRNA 3′ agUGCGCUCGGCUUGCUUGUuu 5′	1073–1092	104.00
		| ||| |:: ||| |||		
		Target 5′ gcAGGCGTGT—GACGCACAtc 3′		

### Quantitative RT-PCR (qRT-PCR)

qRT-PCR was performed as previously described [2]. In brief, an equal amount of total RNA was reverse-transcribed with SuperScript VILO cDNA Synthesis kit (Life Technologies, 11754050) according to manufacturer’s instruction, and diluted with RNase free water and Express SYBR GreenER qPCR Supermix (Life Technologies, 11784–200). Primers we used in this study are listed in Table 2. The relative quantitative analysis was performed by Relative Expression Software Tool (REST) 2009 [24]. Expression values were normalised to the geometric mean of 3 reference genes including *18SrRNA*, *GAPDH*, *β-Tubulin*.

**Table 2:** Information on primers used for qRT-PCR validation.

Primers	Forward (5′->3′)	Reverse (5′->3′)
*Cyclin D2*	GAGTGGGAACTGGTAGTGTTG	CGCACAGAGCGATGAAGGT
*Casp 9*	TCCTGGTACATCGAGACCTTG	AAGTCCCTTTCGCAGAAACAG
*Igf*	CTGGACCAGAGACCCTTTGC	GGACGGGGACTTCTGAGTCTT
*Irak*	AGCCGAGGTCTGCATTACATT	TGGCAGTCTGGATAACTGATGA
*Calm1*	TGGGAATGGTTACATCAGTGC	CGCCATCAATATCTGCTTCTCT
*Calm2*	ACGGGGATGGGACAATAACAA	TGCTGCACTAATATAGCCATTGC
*Jak2*	GAACCTACAGATACGGAGTGTCC	CAAAATCATGCCGCCACT
*Gs*	TGAACAAAGGCATCAAGCAAATG	CAGTCCAGGGTACGGGTCTT
*Gfap*	TCCTTCCAAGGTTGTCCATC	CCAATCAGCCTCAGAGAAGG
*18srRNA*	GCAATTATTCCCCATGAACG	GGGACTTAATCAACGCAAGC
*Β-Tub*	GATCGGTGCTAAGTTCTGGGA	AGGGACATACTTGCCACCTGT
*Gapdh*	AAGATGGTGATGGGCTTCCCG	TGGCAAAGTGGAGATTGTTGCC

### Western Blot

Western blot was performed as previously described [2]. Briefly, proteins were extracted from each retina then the quantity was assessed by QuantiPro BCA assay kit (Sigma-Aldrich, QPBCA). Equal amounts of protein were loaded into NuPage Bis-Tris gels (Life Technologies, NP3023BOX), and transferred to a polyvinylidene difluoride membrane. The membranes were blocked with 5% BSA in TBST and the primary antibodies were incubated overnight at 4°C. Primary antibodies included cyclin D2 (cell signalling, #2924), cleaved caspase 9 (cell signalling, #9509S), calmodulin (CALM, abcam, ab45689), insulin-like growth factor 1 receptor (IGF1r, abcam, ab39398), *glial fibrillary acidic protein* (GFAP, abcam, ab53554) and janus kinase 2 (Jak2, abcam, ab39636). Protein bands were visualised after incubation with the corresponding secondary antibodies conjugated with *horseradish peroxidase*. GeneTool image scanning and analysis software package was used to perform protein bands densitometry and the results were normalised with loading control α/β tubulin (Cell Signalling, #2148).

### Immunofluorescence

Immunofluorescence labelling was performed as previously described [2]. Briefly, eyes were enucleated and anterior segments were dissected immediately. The remaining eye cups were fixed with 4% paraformaldehyde in PBS for an hour at room temperature and cryo-protected with 20% sucrose in PBS for overnight at 4°C. The eyecups were embedded with Optimum Cutting Medium and sectioned at 12μm on Superfrost glass slides. Sections were washed with PBS 3 times for 5 minutes and blocked with normal goat serum for 1 hour at room temperature. After washing with PBS, sections were incubated with primary antibody for overnight at 4°C. The primary antibodies used in this study were cleaved caspase 9 (cell signalling, #9509S), cyclin D2 (cell signalling, #2924), JAK2 (abcam, ab39636) and calmodulin (abcam, ab45689). Secondary antibody incubation was followed on the next day for 2 hours at room temperature and nucleus staining was performed with Hoechst. Stained sections were examined by confocal microscopy as described previously [2, 6].

### *In Situ* Hybridisation

In situ hybridisation was performed to localise miRNA expression in the retina using Dig-labelled miR-133a-3p miRCURY LNA probe (Exiqon, Cat#39270–01) according to the manufacturer’s instruction with minor modifications. Briefly, cryosections were thawed, air-dried and washed with PBS for 3 times. Sections were treated with proteinase K (100ng in 200ml water) in a slide wash container for 5mins followed by acetylation treatment (2.3ml of triethanolamine and 500μl acetic anhydride in 200ml water) for 10mins. A hybridisation buffer containing 25nM of probe was pre-warmed for 5min at 65°C and then applied onto sections. Sections were cover-slipped and incubated in a chamber humidified with 50% formamide in saline sodium citrate (SSC) solution overnight at 53°C. On the next day, sections were washed, blocked with 10% normal goat serum and incubated with an anti-Dig antibody (Roche, 11093274910) overnight at 4°C. NBT/BCIP alkaline phosphatase colour reaction (Vector laboratories, SK-5400) was performed to visualise the cellular localisation of miR-133a expression according to the manufacturer’s instruction.

### Luciferase Assay

Luciferase assay was performed to validate the direct correlation between miRNA and its predicted target gene expression. We chose to validate miR-133a-3p and one of its target genes, cyclin D2, since cyclin D2 was the most differentially expressed gene after Muller cell ablation. Briefly, 3’UTR of cyclin D2 was amplified by PCR, digested with XhoI and NotI restriction enzymes (New England Biolabs, R0146S and R3189S) and cloned into a multiple cloning site located downstream of the renilla translational stop codon within the psiCHECK^TM^-2 vector (Promega, C8021). 661w photoreceptor cells were seeded in 12-well plates (50,000 cells per well) and cultured for 2 days. Once they reached 70% confluence, the cells were transfected with the vector alone, or co-transfected with a miRNA-133a-3p mimic (Ambion, 4464067) or a control miRNA (Ambion; 4464058) using Lipofectamin 3000 (Life Technologies, L3000001) for 48 hrs. Luciferase assay was performed using the Dual-Glo Luciferase Assay System (Promega, E2920) and the luciferase activity was measured by Safire2TM Tecan plate reader (Tecan Austria, Safire-BASIC). Firefly luciferase activity was normalised to the renilla luciferase activity.

### Statistic Analysis

Results are expressed as mean ± SEM. Data were analysed using unpaired Student t-test. A p value <0.05 was regarded as statistically significant.

## Results

### miRNA PCR Array

**Fig 1:**
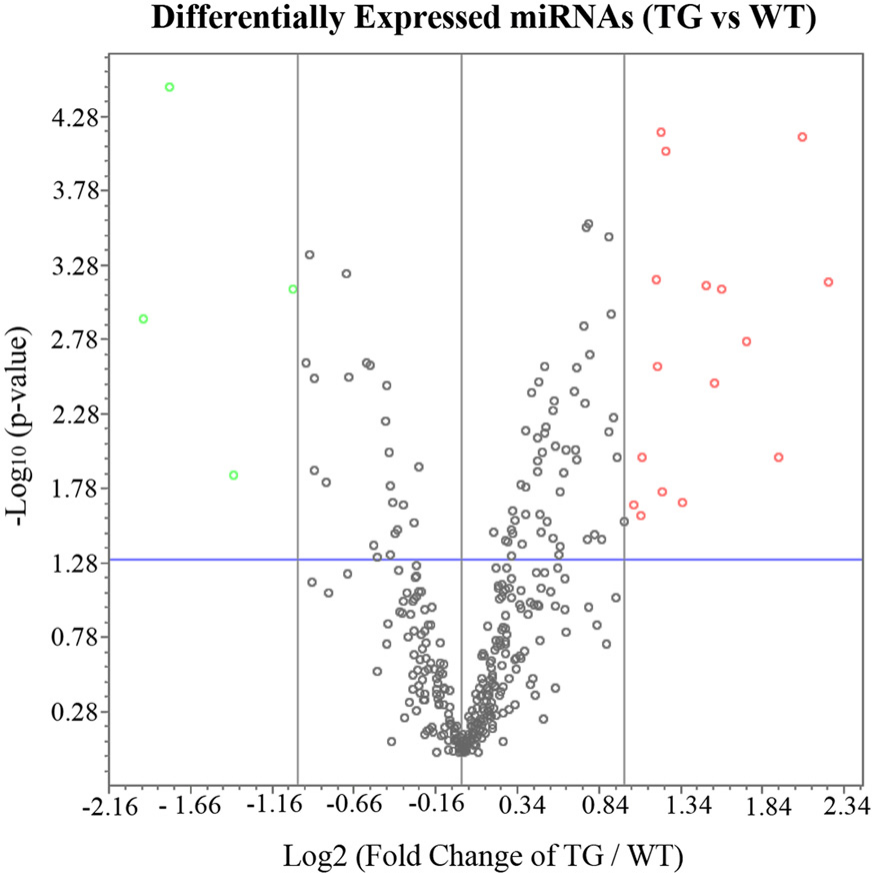
Volcano Plot of Differentially Expressed miRNA in after Selective Müller cell ablation. The red circles represent upregulated miRNAs and the green circles indicate downregulated miRNAs (2 fold-change). The blue line indicates p-value of 0.05.

miRNA PCR array analysis revealed 20 differentially expressed miRNAs with greater than 2 fold change (P < 0.05) 2 weeks after Müller cell ablation. Of these, 16 were upregulated (miR-1196–5p, miR-133a-3p, miR-142–3p, miR-142–5p, miR-146a-5p, miR-146b-3p, miR-146b-5p, miR-190a-5p, miR-1a-1–5p, miR-1a-3p, miR-200a-3p, miR-222–5p, miR-29b-3p, miR-335–3p, miR-335–5p, and miR-653–5p) and 4 were downregulated (miR-1983, miR-375–3p, miR-376c-3p, and miR-542–5p) (Table 3 and Fig. 1). A literature search based functional analysis revealed that the potential functions of the differentially expressed miRNAs were generally associated with pathways contributing to neuronal damage, such as neuronal inflammation and neuronal apoptosis (Table 3). Some of the miRNAs that we found to be differentially regulated after Müller cell ablation have been reported in retinal diseases such as diabetic retinopathy and retinitis pigmentosa (Table 3). In general, the results we obtained from this miRNA PCR array were consistent with our previous studies which identified retinal morphological changes, microarray profiling, neuroinflammation and differential expression of neurotrophins after selective Müller cell ablation [2, 6, 7].

**Table 3:** List of Differentially Expressed miRNAs 2 weeks after induced Müller Cell Ablation.

miRNA	Fold Change	P-value	Potential Functions	References
mmu-miR-1196–5p	2.14	0.0255	Not well characterised	N/A
mmu-miR-133a-3p	2.15	0.0103	Inflammation, Retinitis pigmentosa, Retinal degeneration	[21, 23, 25]
mmu-miR-142–3p	4.26	0.00072	Neuroinflammation, Neurodegeneration, Retinitis Pigmentosa, Diabetes, Retinal degeneration	[21–23, 27, 52, 53]
mmu-miR-142–5p	4.75	0.000685	Neuroinflammation, Autoimmune uvities, Retinitis pigmentosa, Retinal degeneration	[21, 23, 27, 53, 54]
mmu-miR-146a-5p	3.02	0.000072	Neuroinflammation, Diabetes, Neurodegeneration	[22, 26–28, 53, 55, 56]
mmu-miR-146b-3p	3.84	0.01025	Neuroinflammation, Neuronal diseases	[27, 28]
mmu-miR-146b-5p	2.83	0.000728	Neuroinflammation, Neuronal diseases	[27, 28]
mmu-miR-190a-5p	2.08	0.02156	Not well characterised	N/A
mmu-miR-1983	-3.46	0.000033	Not well characterised	N/A
mmu-miR-1a-1–5p	3.35	0.001722	Retinitis pigmentosa, Retinal degeneration	[21, 23]
mmu-miR-1a-3p	2.29	0.00252	Retinitis pigmentosa, Retinal degeneration	[21, 23]
mmu-miR-200a-3p	2.55	0.020964	Neuroinflammation, Neurodegeneration, Diabetes	[22, 25, 26, 28, 29, 52]
mmu-miR-222–5p	2.35	0.017673	Neurnal diseases	[28]
mmu-miR-29b-3p	2.38	0.000091	Diabetes, Neuroinflammation, Neurodegeneration, Neuronal development, Neuronal apoptosis	[18, 26, 28, 55, 57]
mmu-miR-335–3p	2.33	0.000068	Neuroinflammation	[53, 58]
mmu-miR-335–5p	2.29	0.000664	Neuroinflammation	[53, 58]
mmu-miR-375–3p	-2.04	0.000766	Diabetes	[59]
mmu-miR-376c-3p	-2.63	0.01363	Neuroinflammation, Neuronal development	[53]
mmu-miR-542–5p	-3.86	0.001219	Pro-Apoptotic	[60]
mmu-miR-653–5p	2.92	0.003296	Not well characterised	N/A

### Target Gene Analysis and qRT-PCR Validation

**Fig 2:**
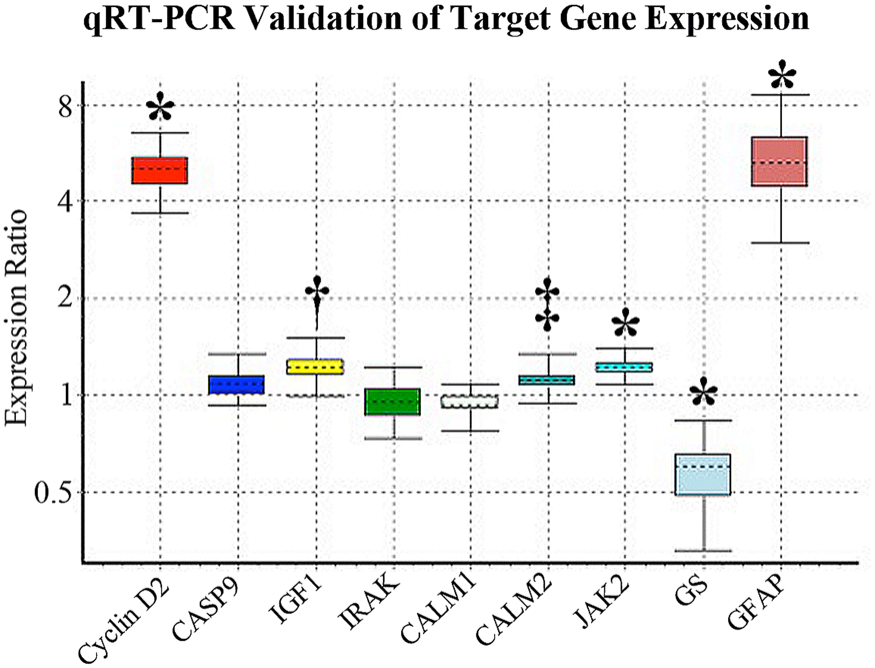
qRT-PCR validation of target gene expression. The boxes represent the interquartile range, and the dotted line represents the median gene expression. Whiskers indicate the maximum and minimum values. * indicates p<0.001, † represents p<0.01 and ‡ shows p<0.05.

We used 2 different databases, TargetScan and mirTarBase, to analyse genes which could be potentially targeted by the differentially expressed miRNAs. We identified 78 overlapping genes that could be potentially targeted by the 20 differentially expressed miRNAs. Of these, 72 corresponded to upregulated miRNAs and 6 to downregulated miRNAs (Table B and Table C in S1 File). DAVID and KEGG pathway analysis revealed that 41 target genes had significant biological functions (Table D in S1 File). Based on their potential roles in retinal pathology as previously reported [2, 6], we chose 7 genes for qRT-PCR validation, including *Cyclin D2*, *Caspase 9*, *IGF1*, *IRAK*, *CALM1*, *CALM2*, and *Jak2* (Table 3). Prior to further validation with qRT-PCR, we performed in silico study with mirTarBAse which revealed seed sequence alignments between miRNAs and their target genes (Table 1). Glutamine synthetase (*GS*) and *GFAP* were used as Müller cell markers to correlate changes in these 7 genes with Müller cell disruption. Consistent with our previous studies [6, 7], Müller cell ablation resulted in downregulation of *GS* and upregulation of *GFAP* (Table 3). qRT-PCR analysis revealed that *Cyclin D2*, *IGF1*, *CALM2* and *Jak2* were significantly upregulated 2 weeks after Müller cell ablation (p<0.05) (Table 4 and Fig. 2). The KEGG pathway analysis suggested that these genes were involved in cell apoptosis, p53, neurotrophin, calcium, chemokine and Jak-STAT signalling pathways (Table 4).

**Table 4:** Summary of some miRNA target genes and signalling pathways that might be involved in the retinal pathology caused by selective Müller cell ablation.

Gene Name	KEGG Pathways/Markers	qRT-PCR Expression	P-value	qRT-PCR Result
*CyclinD2*	p53 Signalling pathway,	5.047	0.001	UP
*Casp9*	Apoptosis, p53 Signalling pathway,	1.081	0.101	-
*Igf1*	p53 Signalling pathway	1.220	0.003	UP
*Irak*	Apoptosis, Neurotrophin pathway	0.950	0.426	-
*Calm1*	Calcium signalling pathway, Neurotrophin pathway	0.932	0.110	-
*Calm2*	Calcium signalling pathway, Neurotrophin pathway	1.109	0.012	UP
*Jak2*	Chemokine Signalling pathway, Jak-STAT Signalling Pathway	1.222	0.001	UP
*GS*	Müller Cell Marker	0.561	0.000	DOWN
*Gfap*	Gliosis Marker	5.277	0.001	UP

### Western Blot Analysis

**Fig 3:**
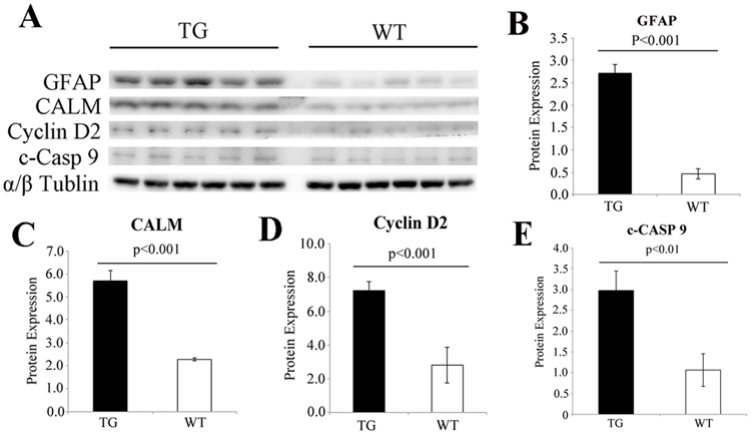
Western blot validation of target gene expression. (A-E) Western blot analysis revealed significant upregulation of proteins related to gliosis, calcium signalling pathway and p53 signalling pathways. The results were consistent with the qRT-PCR analysis. GFAP; glia fibrillary acidic protein, CALM; calmodulin, c-CASP 9; cleaved caspase 9.

Western blot analysis after Müller cell ablation found changes that were consistent with target gene analysis revealed by qRT-PCR. Genes involved in the p53 signalling pathway, cell apoptosis including cleaved-caspase 9 and cyclin D2 and calmodulin which plays a role in calcium signalling and the neurotrophin pathway, were all significantly upregulated after Müller cell ablation (Fig. 3). Jak2 tended to be upregulated but the change was not statistical significant (P = 0.2).

### Immunofluorescence

**Fig 4:**
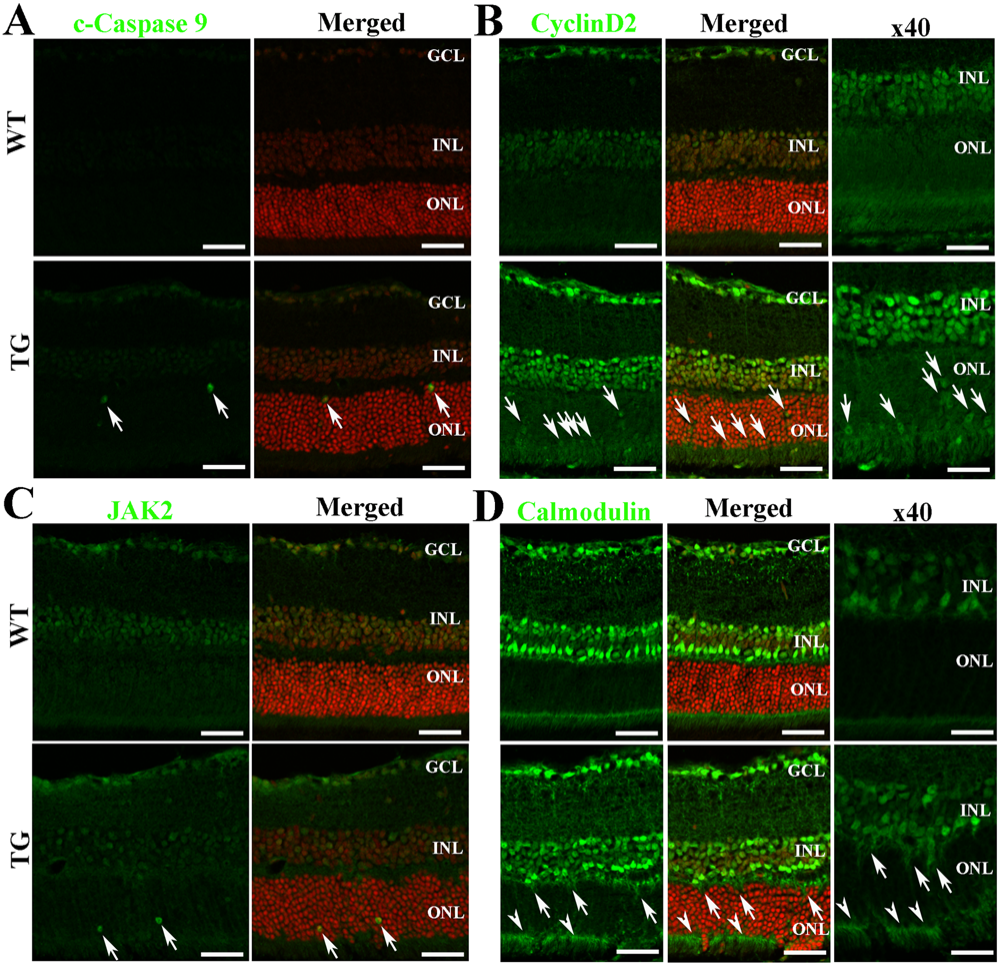
Immunofluorescent staining for proteins encoded by genes targeted by differentially expressed miRNAs after Müller cell ablation. The genes encoding C-caspase 9 and CyclinD2 are targeted by miR133a-3p. “Merged” includes Hoechst stain. Jak2 is encoded by a gene that is targeted by miR-375–3p. Calmodulin is a gene targeted by miR1a-1. Cleaved-caspase 9 (A, arrows) and Jak2 (C, arrows) were only observed after Müller cell ablation (A and C). Increased immunoreactivity for CyclinD2 was observed in the ganglion cell layer (GCL), inner nuclear layer (INL) and outer nuclear layer (ONL) after Müller cell ablation compared with controls (B). Calmodulin was mainly expressed in the GCL and retinal neurons in the INL in the normal retina but increased expression (arrows) was observed in the outer plexiform layer, ONL and photoreceptor segments (arrow heads) in areas of Müller cell ablation, which are marked by focal areas of degeneration in the ONL (D). Scale bars represent 50μm. Scale bars in figures with high magnification (x40 in B and D) represent 25μm.

We performed immunofluorescence with antibodies against four proteins encoded by target genes including cleaved-caspase 9, cyclin D2, Jak2 and calmodulin to examine their cellular specific expression (Fig. 4). We found that cleaved-caspase9 (Fig. 4A) and Jak2 (Fig. 4C) were mainly expressed in the outer nuclear layer (ONL) in areas corresponding with patches of Müller cell ablation. Upregulation of cyclin D2 was observed in not only the inner nuclear layer (INL) and the ONL but also in some cell bodies of photoreceptors near the outer limiting membrane after Müller cell ablation compared with controls (Fig. 4B). Calmodulin was mainly expressed in the INL and weakly expressed in photoreceptor inner segments in the normal retinae. It was markedly upregulated in the outer plexiform layer, ONL and photoreceptor inner segments in areas of Müller cell ablation (Fig. 4D).

### Validation of the Correlation of miR-133a-3p with Its Target Gene Cyclin D2 Expression Using Luciferase Assay

**Fig 5:**
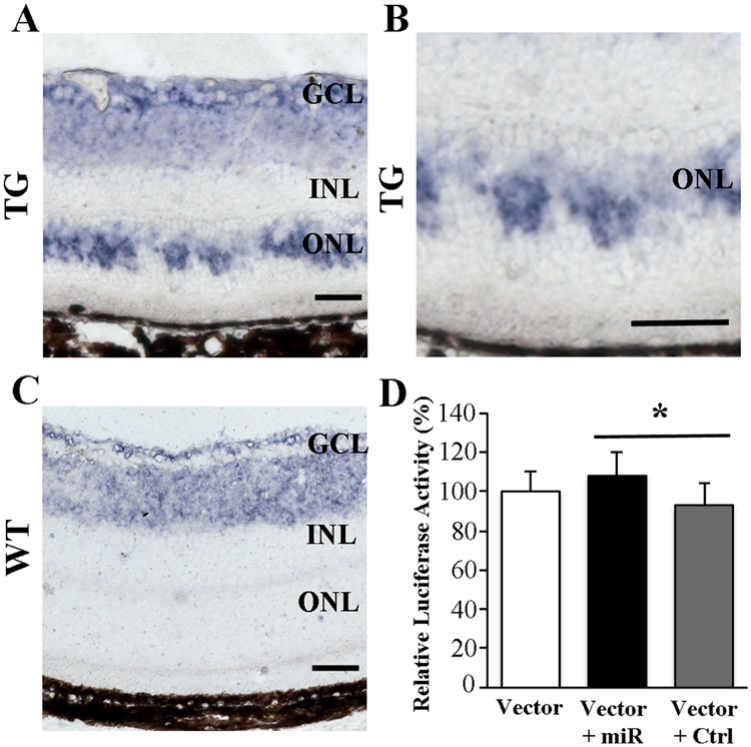
(A-C), in situ hybridisation for miR-133a-3p expression in normal (C) and diseased (A and B) retinas. (D), luciferase assay to study the correlation of miR-133a-3p with cyclin D2 expression in 661 photoreceptor cells. The expression of miR-133a-3p in the outer nuclear layer (ONL) was increased after induced Müller cell ablation. Co-transfection of 661w cells using a vector containing 3’UTR region of the cyclin D2 gene along with a miR-133a-3p mimic (Vector + miR) induced a significant increase in the normalised firefly luciferase activity compared with cells co-transfected using the vector with a control miRNA (Vector + Ctrl) (*P<0.05, n = 6/group). Data is presented as Mean + SEM. Scale bars in A-C: 50μm. GCL = ganglion cell layer. INL = inner nuclear layer. ONL = outer nuclear layer.

miRNA PCR array showed 2.15-fold increase in miR-133a-3p expression (Table 3) while qRT-PCR analysis showed 5-fold increase in cyclin D2 expression after Muller cell ablation (Table 4). As cyclin D2 is one of the genes targeted by miR-133a-3p, our data indicate a positive correlation between miR-133a-3p and cyclin D2 expression. We performed *in situ* hybridisation and found that the increased miR-133a-3p expression was mainly localised to the outer nuclear layer in the retina after Muller cell ablation (Fig. 5A-C). In order to validate the functional correlation between miR-133a-3p and cyclin D2 expression, we cloned the 3’UTR region of cyclin D2 gene into a vector and conducted luciferase assay in 661w photoreceptor cells. We found that co-transfection of 661w cells with a miR-133a-3p mimic along with the vector resulted in significant increase in the normalised luciferase activity compared with cells co-transfected with a control miRNA and the vector (P<0.05, Fig. 5D), thus confirming the positive correlation between miR-133a-3p and cyclin D2 expression.

## Discussion

We have identified 20 miRNAs that were differentially expressed during the photoreceptor degeneration that occurred after selective Müller cell ablation. We then used 2 different databases, TargetScan and mirTarBase, to identify a number of genes that could be potentially targeted by these miRNAs. Of 72 target genes that we identified, 41 were found, by DAVID and KEGG pathway analysis, to be involved in several biological functions. Based on our previous studies, we chose 7 target genes which were involved in apoptosis, p53, chemokine, Jak-STAT, calcium and neurotrophin signalling pathways for further validation. Upregulation of the chosen target genes was confirmed with qRT-PCR and validated by western blot analysis. Immunofluorescence studies revealed that the upregulation of these miRNA-target genes were mainly observed in the ONL where photoreceptor degeneration occurred after selective Müller cell ablation. We conducted in situ hybridisation for miR-133a-3p and found that the increased expression of miR-133a-3p was mainly localised in the ONL. Furthermore, we performed luciferase assay to validate the positive correlation between miR133a-3p and one of its predicted targets genes, cyclin D2 in 661 photoreceptor cells. The results were generally consistent with our previous findings that selective Müller cell ablation causes photoreceptor degeneration [2, 6].

Neuronal damage in this transgenic model is characterized by photoreceptor degeneration caused by selective Müller cell ablation [6]. MiRNA profiling conducted 2 weeks after Müller cell ablation identified a number of differentially expressed miRNAs whose target genes were associated with cell apoptosis and neurodegeneration in the central nervous system including retinal degeneration [21, 23, 25–29]. For instance, one of the genes targeted by miR-133a-3p is caspase-9, which can form an apoptosome with cytochrome-c released from mitochondria and lead to activation of other caspases and cell death [30]. Calmodulin, which is targeted by mir-1a-3, has been reported that calmodulin plays a role in neurotransmitter release and trafficking in synapsis [31, 32]. However, overexpression of calmodulin has been reported to be involved in neuronal apoptosis by activating calcium/calmodulin-dependent protein kinase II [33, 34]. Inhibition of calmodulin activity has been reported to prevent retinal neuronal apoptosis [35–38].

Our previous studies found that the microarray profiling in which we performed 1 week after Müller cell ablation, also revealed upregulation of genes involved in apoptosis [2], and the characterization study showed photoreceptor apoptosis was peaked at 2 weeks after Müller cell ablation [6]. We did not observe any significant differences in the number of TUNNEL positive cells the retina between 1 week and 2 weeks after Müller cell ablation, thus we believe the current study is generally supported by our previously observations.

Interestingly, miR-133a-3p also targets an anti-apoptotic gene. Cyclin D2, which has been reported to play a role in anti-apoptosis by phosphorylating retinoblastoma protein, cell cycle arrest and neurogenesis[39–42], was significantly upregulated after Müller cell ablation. After confirmation that the increased miR-133a-3p expression was mainly localised to the outer nuclear layer after Muller cell ablation, we conducted luciferase assay in 661w photoreceptors to validate the positive correlation between miR-133a-3p and cyclin D2 expression. We found that co-transfection of 661w cells using the vector with a miR-133a mimic induced significant increase in luciferase activity compared with cells co-transfected using the vector with a control miRNA (Fig. 5D). A previous study reported a negative correlation between miR-133a and cyclin D2 expression in cardiac muscle cells and cos-1 cells [41]. The discrepancy between that study and the results we present here could be due to the diverse roles of miR-133a and cyclin D2 in different tissues and biological models. Cyclin D family has been reported to play a neuroprotective role in brain injury and retinal degeneration [41, 43]. Moreover, cyclin D2 is involved in neurogenesis [40]. It is unclear whether the overexpressed miR-133a and cyclin D2 are due to a defensive response to the retinal stress caused by Muller cell ablation. Future studies are warranted to investigate the effects of intervention of miR-133a and cyclin D2 overexpression on photoreceptor degeneration in our transgenic mice.

Neuroinflammation was also shown in the transgenic model of selective Müller cell ablation [2, 6, 7]. Mir-146a-5p, which has been reported to play a role in neuroinflammation and neurodegeneration [22, 27], was upregulated after induced Müller cell ablation. It has been reported that its target genes, such as IRAK1, IRAK2, and TRAF6, are involved in cell survival and neuroprotection. IRAK recruits the p75 neurotrophin receptor and activates nuclear factor keppa B (NF-kB) [44, 45]. We recently showed that the p75 neurotrophin receptor was profoundly upregulated in activated surviving Müller cells and that photoreceptor degeneration occurred concurrently with activation of microglial cells in this model. Linking the result of upregulation of mir-146a-5p with features of photoreceptor degeneration and neuroinflammation caused by Müller cell ablation indicates a potential positive correlation between mir-146a-5p expression and IRAK expression, although our qRT-PCR analysis of the change in IRAK transcription was not statistically significant between transgenic and control mice. Further analysis on the relationship between miR-146a-5p upregulation of changes in different isoforms of IRAKs and their downstream effectors is warranted to provide a better understanding of the role of miRNA-146 in photoreceptor degeneration and neuroinflammation caused by Müller cell dysfunction.

Interestingly, miR-375–3p was downregulated after Müller cell ablation, and Jak2, one of its target genes, was upregulated. The negative correlation between miR-375 and Jak2 is consistent with a recent report in which target mutation of a predicted miR-375-binding site abolished the activity of a luciferase reporter carrying the 3’ untranslated region of Jak2 [46]. Jak2 has been reported to be involved in activation of immune response/chemokine signalling and the Jak/STAT pathways. Inflammation induced by lipopolysachrides in the rat retina caused activation of the Jak2/STAT3 pathway and neuronal gliosis [47]. Our recent studies found that selective Müller cell ablation resulted in profound activation of surviving Müller cells and microglia and overexpression of TNFα. It is plausible that the downregulation of miR-375–3p may trigger activation of the Jak2/STAT3 pathway and chemokine signalling, both of which can damage photoreceptors.

We conducted immunostaining to study the cellular localization of a number of target genes in normal and degenerating retinae. Cleaved-caspase 9, cyclin D2 and JAK2, were mainly expressed in the ONL in areas corresponding to Müller cell ablation, whereas upregulation of calmodulin was observed in the outer plexiform layer, ONL and photoreceptor segments after Müller cell ablation. Calmodulin is a highly conserved regulatory protein found in all eukaryotic organisms which mediates a variety of calcium ion-dependent signaling pathways [31]. A recent study showed that over-activation of calcium/calmodulin-activated protein kinase II is involved in photoreceptor degeneration in the rd1 mouse [48]. Our observation that calmodulin was upregulated after Müller cell ablation suggests that activation of the calcium/calmodulin signalling may contribute to photoreceptor degeneration. Interestingly, cyclin D2 was highly upregulated in the INL of retinae with Müller cell ablation compared with the normal retina in controls. As cyclin D2 is anti-apoptosis [39], this observation may partially explain the phenomenon that neuronal damage is not obvious in the INL following Müller cell ablation in this transgenic model [6].

One of the interesting findings we found in this study is that a number of differentially expressed miRNAs appear to be positively correlated with their target gene expression after selective Müller cell ablation. While the precise molecular mechanisms of which miRNA control gene expression are still largely obscure, there has been a growing number of reports of a positive correlation between miRNA and mRNA expression [17, 49–51]. Some hypothetical and *in silico* studies of the mechanism of which miRNA promotes gene expression have recently been reported [14–17, 50].

We acknowledge that this profiling study only covered 372 miRNAs rather than an unbiased genome-wide screen. We cannot rule out the possibility that the screening of a limited number of miRNAs might have missed some miRNAs which make important contributions towards photoreceptor degeneration and neuroinflammation in retinal disease.

We report here that miRNAs and their target genes that are differentially expressed soon after selective Müller ablation are mainly involved in neuronal apoptosis, inflammation and neurodegenration. Müller cell dysfunction has been implicated in the pathogenesis of many retinal diseases but its precise contribution to the photoreceptor degeneration, which ultimately results in loss of vision, remains poorly understood. The results of miRNA profiling after Müller cell ablation that we present here provide further insights into the pathogenesis of photoreceptor degeneration associated with Müller cell dysfunction.

## Supporting Information

S1 File Supporting tables. Table A. The list of 372 miRNA IDs used in Qiagen HC PCR array. Table B. Target gene analysis from the upregulated miRNAs. The list shows the overlapping target genes from TargetScan and miTarBase. Table C. Target gene analysis from the dwonregulated miRNAs. The list is the overlapping target genes from TargetScan and miTarBase. Table D. Functional analysis of the target genes performed by DAVID and KEGG.
(DOCX)

## References

[1] Jablonski MM, Iannaccone A (2000) Targeted disruption of Muller cell metabolism induces photoreceptor dysmorphogenesis. Glia 32: 192–204. 11008218 10.1002/1098-1136(200011)32:2<192::aid-glia80>3.0.co;2-6

[2] Chung SH, Shen W, Gillies MC (2013) Laser capture microdissection-directed profiling of glycolytic and mTOR pathways in areas of selectively ablated Muller cells in the murine retina. Investigative ophthalmology & visual science 54: 6578–6585. 23982838 10.1167/iovs.13-12311

[3] Tout S, Chan-Ling T, Hollander H, Stone J (1993) The role of Muller cells in the formation of the blood-retinal barrier. Neuroscience 55: 291–301. 8350991 10.1016/0306-4522(93)90473-s

[4] Powner MB, Gillies MC, Tretiach M, Scott A, Guymer RH, Hageman GS, et al (2010) Perifoveal muller cell depletion in a case of macular telangiectasia type 2. Ophthalmology 117: 2407–2416. 10.1016/j.ophtha.2010.04.001 20678804 PMC2974049

[5] Rungger-Brandle E, Dosso AA PM (2000) Glial reactivity, an early feature of diabetic retinopathy. Investigative ophthalmology & visual science 41: 1971–1980. 10845624

[6] Shen W, Fruttiger M, Zhu L, Chung SH, Barnett NL, Kirk JK, et al (2012) Conditional Muller Cell Ablation Causes Independent Neuronal and Vascular Pathologies in a Novel Transgenic Model. The Journal of neuroscience: the official journal of the Society for Neuroscience 32: 15715–15727. 23136411 10.1523/JNEUROSCI.2841-12.2012 PMC4014009

[7] Shen W, Zhu L, Lee SR, Chung SH, Gillies MC (2013) Involvement of NT3 and P75NTR in photoreceptor degeneration following selective Muller cell ablation. Journal of neuroinflammation 10: 137 10.1186/1742-2094-10-137 24224958 PMC3831588

[8] Sundermeier TR, Palczewski K (2012) The physiological impact of microRNA gene regulation in the retina. Cellular and molecular life sciences: CMLS 69: 2739–2750. 10.1007/s00018-012-0976-7 22460583 PMC3443797

[9] Ambros V (2004) The functions of animal microRNAs. Nature 431: 350–355. 15372042 10.1038/nature02871

[10] Huntzinger E E (2011) Gene silencing by microRNAs: contributions of translational repression and mRNA decay. Nature reviews Genetics 12: 99–110. 10.1038/nrg2936 21245828

[11] Fabian MR, Sonenberg N, Filipowicz W (2010) Regulation of mRNA translation and stability by microRNAs. Annual review of biochemistry 79: 351–379. 10.1146/annurev-biochem-060308-103103 20533884

[12] Fabian MR, Sundermeier TR, Sonenberg N (2010) Understanding how miRNAs post-transcriptionally regulate gene expression. Progress in molecular and subcellular biology 50: 1–20. 10.1007/978-3-642-03103-8_1 19841878

[13] Siomi H, Siomi MC (2010) Posttranscriptional regulation of microRNA biogenesis in animals. Molecular cell 38: 323–332. 10.1016/j.molcel.2010.03.013 20471939

[14] Vasudevan S, Tong Y, Steitz JA (2007) Switching from repression to activation: microRNAs can up-regulate translation. Science 318: 1931–1934. 18048652 10.1126/science.1149460

[15] Vasudevan S (2012) Posttranscriptional upregulation by microRNAs. Wiley interdisciplinary reviews RNA 3: 311–330. 10.1002/wrna.121 22072587

[16] Lee S, Vasudevan S (2013) Post-transcriptional stimulation of gene expression by microRNAs. Advances in experimental medicine and biology 768: 97–126. 10.1007/978-1-4614-5107-5_7 23224967

[17] Place RF, Li LC, Pookot D, Noonan EJ, Dahiya R (2008) MicroRNA-373 induces expression of genes with complementary promoter sequences. Proceedings of the National Academy of Sciences of the United States of America 105: 1608–1613. 10.1073/pnas.0707594105 18227514 PMC2234192

[18] Hackler L Jr, Wan J, Swaroop A, Qian J, Zack DJ (2010) MicroRNA profile of the developing mouse retina. Investigative ophthalmology & visual science 51: 1823–1831. 19933188 10.1167/iovs.09-4657 PMC2868396

[19] Karali M, Peluso I, Gennarino VA, Bilio M, Verde R, Lago G, et al (2010) miRNeye: a microRNA expression atlas of the mouse eye. BMC genomics 11: 715 10.1186/1471-2164-11-715 21171988 PMC3018480

[20] Krol J, Busskamp V, Markiewicz I, Stadler MB, Ribi S, Richter J, et al (2010) Characterizing light-regulated retinal microRNAs reveals rapid turnover as a common property of neuronal microRNAs. Cell 141: 618–631. 10.1016/j.cell.2010.03.039 20478254

[21] Loscher CJ, Hokamp K, Wilson JH, Li T, Humphries P, Farrar GJ, et al (2008) A common microRNA signature in mouse models of retinal degeneration. Experimental eye research 87: 529–534. 10.1016/j.exer.2008.08.016 18834879 PMC4030402

[22] Kovacs B, Lumayag S, Cowan C, Xu S (2011) MicroRNAs in early diabetic retinopathy in streptozotocin-induced diabetic rats. Investigative ophthalmology & visual science 52: 4402–4409. 21498619 10.1167/iovs.10-6879

[23] Loscher CJ, Hokamp K, Kenna PF, Ivens AC, Humphries P, Palfi A, et al (2007) Altered retinal microRNA expression profile in a mouse model of retinitis pigmentosa. Genome biology 8: R248 18034880 10.1186/gb-2007-8-11-r248 PMC2258196

[24] Pfaffl MW, Horgan GW L (2002) Relative expression software tool (REST) for group-wise comparison and statistical analysis of relative expression results in real-time PCR. Nucleic Acids Res 30: e36 11972351 10.1093/nar/30.9.e36 PMC113859

[25] Freilich RW, Woodbury ME, Ikezu T (2013) Integrated Expression Profiles of mRNA and miRNA in Polarized Primary Murine Microglia. PloS one 8: e79416 10.1371/journal.pone.0079416 24244499 PMC3823621

[26] Goodall EF, Heath PR, Bandmann O, Kirby J, Shaw PJ (2013) Neuronal dark matter: the emerging role of microRNAs in neurodegeneration. Frontiers in cellular neuroscience 7: 178 10.3389/fncel.2013.00178 24133413 PMC3794211

[27] Ksiazek-Winiarek DJ, Kacperska MJ, Glabinski A (2013) MicroRNAs as novel regulators of neuroinflammation. Mediators of inflammation 2013: 172351 10.1155/2013/172351 23983402 PMC3745967

[28] Maes OC, Chertkow HM, Wang E, Schipper HM (2009) MicroRNA: Implications for Alzheimer Disease and other Human CNS Disorders. Current genomics 10: 154–168. 10.2174/138920209788185252 19881909 PMC2705849

[29] McArthur K, Feng B, Wu Y, Chen S, Chakrabarti S (2011) MicroRNA-200b regulates vascular endothelial growth factor-mediated alterations in diabetic retinopathy. Diabetes 60: 1314–1323. 10.2337/db10-1557 21357793 PMC3064105

[30] Fridman J, SLowe SW (2003) Control of apoptosis by p53. Oncogene 22: 9030–9040. 14663481 10.1038/sj.onc.1207116

[31] DeLorenzo RJ (1982) Calmodulin in neurotransmitter release and synaptic function. Federation proceedings 41: 2265–2272. 6122609

[32] Hinds HL, Goussakov I, Nakazawa K, Tonegawa S, Bolshakov VY (2003) Essential function of alpha-calcium/calmodulin-dependent protein kinase II in neurotransmitter release at a glutamatergic central synapse. Proceedings of the National Academy of Sciences of the United States of America 100: 4275–4280. 12629219 10.1073/pnas.0530202100 PMC153083

[33] Ui-Tei K, Nagano M, Sato S, Miyata Y (2000) Calmodulin-dependent and-independent apoptosis in cell of a Drosophila neuronal cell line. Apoptosis: an international journal on programmed cell death 5: 133–140. 11232241 10.1023/a:1009676528805

[34] Yu W, Niwa T, Miura Y, Horio F, Teradaira S, Ribar TJ, et al (2002) Calmodulin overexpression causes Ca(2+)-dependent apoptosis of pancreatic beta cells, which can be prevented by inhibition of nitric oxide synthase. Laboratory investigation; a journal of technical methods and pathology 82: 1229–1239. 12218084 10.1097/01.lab.0000027921.01548.c5

[35] Goebel DJ (2009) Selective blockade of CaMKII-alpha inhibits NMDA-induced caspase-3-dependent cell death but does not arrest PARP-1 activation or loss of plasma membrane selectivity in rat retinal neurons. Brain research 1256: 190–204. 10.1016/j.brainres.2008.12.051 19135986

[36] Fan W, Agarwal N, Kumar MD, Cooper NG (2005) Retinal ganglion cell death and neuroprotection: Involvement of the CaMKIIalpha gene. Brain research Molecular brain research 139: 306–316. 16023257 10.1016/j.molbrainres.2005.06.008

[37] Fan W, Agarwal N, Cooper NG (2006) The role of CaMKII in BDNF-mediated neuroprotection of retinal ganglion cells (RGC-5). Brain research 1067: 48–57. 16337157 10.1016/j.brainres.2005.10.030

[38] Fan W, Cooper NG (2009) Glutamate-induced NFkappaB activation in the retina. Investigative ophthalmology & visual science 50: 917–925. 18836176 10.1167/iovs.08-2555 PMC4446986

[39] Bai M, Tsanou E, Skyrlas A, Sainis I, Agnantis N, Kanavaros P (2007) Alterations of the p53, Rb and p27 tumor suppressor pathways in diffuse large B-cell lymphomas. Anticancer research 27: 2345–2352. 17695524

[40] Kowalczyk A, Filipkowski RK, Rylski M, Wilczynski GM, Konopacki FA, Jaworski J, et al (2004) The critical role of cyclin D2 in adult neurogenesis. The Journal of cell biology 167: 209–213. 15504908 10.1083/jcb.200404181 PMC2172537

[41] Liu N, Bezprozvannaya S, Williams AH, Qi X, Richardson JA, Bassel-Duby R, et al (2008) microRNA-133a regulates cardiomyocyte proliferation and suppresses smooth muscle gene expression in the heart. Genes & development 22: 3242–3254. 19015276 10.1101/gad.1738708 PMC2600761

[42] Urbach A, Robakiewicz I, Baum E, Kaczmarek L, Witte OW, Filipkowski RK (2013) Cyclin D2 knockout mice with depleted adult neurogenesis learn Barnes maze task. Behavioral neuroscience 127: 1–8. 10.1037/a0031222 23244288

[43] Ma C, Papermaster D, Cepko CL (1998) A unique pattern of photoreceptor degeneration in cyclin D1 mutant mice. Proceedings of the National Academy of Sciences of the United States of America 95: 9938–9943. 9707579 10.1073/pnas.95.17.9938 PMC21440

[44] Cao Z, Henzel W, JGao X (1996) IRAK: a kinase associated with the interleukin-1 receptor. Science 271: 1128–1131. 8599092 10.1126/science.271.5252.1128

[45] Mamidipudi V, Li X, Wooten MW (2002) Identification of interleukin 1 receptor-associated kinase as a conserved component in the p75-neurotrophin receptor activation of nuclear factor-kappa B. The Journal of biological chemistry 277: 28010–28018. 12034707 10.1074/jbc.M109730200

[46] Ding L, Xu Y, Zhang W, Deng Y, Si M, Du Y, et al (2010) MiR-375 frequently downregulated in gastric cancer inhibits cell proliferation by targeting JAK2. Cell research 20: 784–793. 10.1038/cr.2010.79 20548334

[47] Jang S, Lee JH, Choi KR, Kim D, Yoo H, SOh S (2007) Cytochemical alterations in the rat retina by LPS administration. Neurochemical research 32: 1–10. 17160463 10.1007/s11064-006-9215-7

[48] Hauck SM, Ekstrom PA, Ahuja-Jensen P, Suppmann S, Paquet-Durand F, van Veen T, et al (2006) Differential modification of phosducin protein in degenerating rd1 retina is associated with constitutively active Ca2+/calmodulin kinase II in rod outer segments. Molecular & cellular proteomics: MCP 5: 324–336. 16253986 10.1074/mcp.M500217-MCP200

[49] Mortensen RD, Serra M, Steitz JA, Vasudevan S (2011) Posttranscriptional activation of gene expression in Xenopus laevis oocytes by microRNA-protein complexes (microRNPs). Proceedings of the National Academy of Sciences of the United States of America 108: 8281–8286. 10.1073/pnas.1105401108 21536868 PMC3100953

[50] Nunez YO, Truitt JM, Gorini G, Ponomareva ON, Blednov YA, Harris RA, et al (2013) Positively correlated miRNA-mRNA regulatory networks in mouse frontal cortex during early stages of alcohol dependence. BMC genomics 14: 725 10.1186/1471-2164-14-725 24148570 PMC3924350

[51] Nunez-Iglesias J, Liu CC, Morgan TE, Finch C, EZhou XJ (2010) Joint genome-wide profiling of miRNA and mRNA expression in Alzheimer’s disease cortex reveals altered miRNA regulation. PloS one 5: e8898 10.1371/journal.pone.0008898 20126538 PMC2813862

[52] Lau P, Bossers K, Janky R, Salta E, Frigerio CS, Barbash S, et al (2013) Alteration of the microRNA network during the progression of Alzheimer’s disease. EMBO molecular medicine 5: 1613–1634. 10.1002/emmm.201201974 24014289 PMC3799583

[53] Jovicic A, Roshan R, Moisoi N, Pradervand S, Moser R, Pillai B, et al (2013) Comprehensive expression analyses of neural cell-type-specific miRNAs identify new determinants of the specification and maintenance of neuronal phenotypes. The Journal of neuroscience: the official journal of the Society for Neuroscience 33: 5127–5137. 10.1523/JNEUROSCI.0600-12.2013 23516279 PMC6705001

[54] Ishida W, Fukuda K, Higuchi T, Kajisako M, Sakamoto S, Fukushima A (2011) Dynamic changes of microRNAs in the eye during the development of experimental autoimmune uveoretinitis. Investigative ophthalmology & visual science 52: 611–617. 20881307 10.1167/iovs.10-6115

[55] Omran A, Elimam D, Yin F (2013) MicroRNAs: new insights into chronic childhood diseases. BioMed research international 2013: 291826 10.1155/2013/291826 23878802 PMC3710618

[56] Feng B, Chen S, McArthur K, Wu Y, Sen S, Ding Q, et al (2011) miR-146a-Mediated extracellular matrix protein production in chronic diabetes complications. Diabetes 60: 2975–2984. 10.2337/db11-0478 21885871 PMC3198068

[57] Silva VA, Polesskaya A, Sousa TA, Correa VM, Andre ND, Reis RI, et al (2011) Expression and cellular localization of microRNA-29b and RAX, an activator of the RNA-dependent protein kinase (PKR), in the retina of streptozotocin-induced diabetic rats. Molecular vision 17: 2228–2240. 21897745 PMC3164688

[58] Cossetti C, Smith JA, Iraci N, Leonardi T, Alfaro-Cervello C, Pluchino S (2012) Extracellular membrane vesicles and immune regulation in the brain. Frontiers in physiology 3: 117 10.3389/fphys.2012.00117 22557978 PMC3340916

[59] Poy MN, Eliasson L, Krutzfeldt J, Kuwajima S, Ma X, Macdonald PE, et al (2004) A pancreatic islet-specific microRNA regulates insulin secretion. Nature 432: 226–230. 15538371 10.1038/nature03076

[60] Bray I, Tivnan A, Bryan K, Foley NH, Watters KM, Tracey L, et al (2011) MicroRNA-542–5p as a novel tumor suppressor in neuroblastoma. Cancer letters 303: 56–64. 10.1016/j.canlet.2011.01.016 21310526 PMC3057396

