# Supplementary material for: Profiling of MicroRNAs Involved in Retinal Degeneration Caused by Selective Müller Cell Ablation
Source: PLoS One. 2015 Mar 5;10(3):e0118949. doi: 10.1371/journal.pone.0118949 (PMC4351074; doi:10.1371/journal.pone.0118949)
Supplement: S1 File — Table A. The list of 372 miRNA IDs used in Qiagen HC PCR array. Table B. Target gene analysis from the upregulated miRNAs. The list shows the overlapping target genes from TargetScan and miTarBase. Table C. Target gene analysis from the dwonregulated miRNAs. The list is the overlapping target genes from TargetScan and miTarBase. Table D. Functional analysis of the target genes performed by DAVID and KEGG. (DOCX) [file pone.0118949.s001.docx]

***Supporting Information Tables***

[Table A] The list of 372 miRNA IDs used in Qiagen HC PCR array.

| **Plate Position** | **miRNA ID** |
| --- | --- |
| A01 | mmu-let-7a-5p |
| A02 | mmu-let-7b-5p |
| A03 | mmu-let-7c-5p |
| A04 | mmu-let-7d-5p |
| A05 | mmu-let-7d-3p |
| A06 | mmu-let-7e-5p |
| A07 | mmu-let-7f-5p |
| A08 | mmu-let-7g-5p |
| A09 | mmu-let-7i-5p |
| A10 | mmu-miR-100-5p |
| A11 | mmu-miR-101a-3p |
| A12 | mmu-miR-101a-5p |
| A13 | mmu-miR-101b-3p |
| A14 | mmu-miR-103-3p |
| A15 | mmu-miR-106a-5p |
| A16 | mmu-miR-106b-5p |
| A17 | mmu-miR-107-3p |
| A18 | mmu-miR-10a-5p |
| A19 | mmu-miR-10b-5p |
| A20 | mmu-miR-122-5p |
| A21 | mmu-miR-124-3p |
| A22 | mmu-miR-124-5p |
| A23 | mmu-miR-125a-5p |
| A24 | mmu-miR-125b-5p |
| B01 | mmu-miR-126a-3p |
| B02 | mmu-miR-126a-5p |
| B03 | mmu-miR-128-3p |
| B04 | mmu-miR-129-5p |
| B05 | mmu-miR-130a-3p |
| B06 | mmu-miR-130b-3p |
| B07 | mmu-miR-132-3p |
| B08 | mmu-miR-133a-3p |
| B09 | mmu-miR-133a-5p |
| B10 | mmu-miR-133b-3p |
| B11 | mmu-miR-134-5p |
| B12 | mmu-miR-135a-5p |
| B13 | mmu-miR-135b-5p |
| B14 | mmu-miR-137-3p |
| B15 | mmu-miR-138-5p |
| B16 | mmu-miR-139-5p |
| B17 | mmu-miR-140-5p |
| B18 | mmu-miR-141-3p |
| B19 | mmu-miR-142-3p |
| B20 | mmu-miR-142-5p |
| B21 | mmu-miR-143-3p |
| B22 | mmu-miR-143-5p |
| B23 | mmu-miR-144-3p |
| B24 | mmu-miR-145a-5p |
| C01 | mmu-miR-145a-3p |
| C02 | mmu-miR-146a-5p |
| C03 | mmu-miR-146b-5p |
| C04 | mmu-miR-146b-3p |
| C05 | mmu-miR-147-3p |
| C06 | mmu-miR-148a-3p |
| C07 | mmu-miR-148b-3p |
| C08 | mmu-miR-149-5p |
| C09 | mmu-miR-150-5p |
| C10 | mmu-miR-151-3p |
| C11 | mmu-miR-151-5p |
| C12 | mmu-miR-152-3p |
| C13 | mmu-miR-153-3p |
| C14 | mmu-miR-155-5p |
| C15 | mmu-miR-155-3p |
| C16 | mmu-miR-15a-5p |
| C17 | mmu-miR-15b-5p |
| C18 | mmu-miR-16-5p |
| C19 | mmu-miR-17-5p |
| C20 | mmu-miR-181a-5p |
| C21 | mmu-miR-181b-5p |
| C22 | mmu-miR-181c-5p |
| C23 | mmu-miR-181d-5p |
| C24 | mmu-miR-182-5p |
| D01 | mmu-miR-182-3p |
| D02 | mmu-miR-183-5p |
| D03 | mmu-miR-183-3p |
| D04 | mmu-miR-184-3p |
| D05 | mmu-miR-185-5p |
| D06 | mmu-miR-186-5p |
| D07 | mmu-miR-187-3p |
| D08 | mmu-miR-18a-5p |
| D09 | mmu-miR-190a-5p |
| D10 | mmu-miR-191-5p |
| D11 | mmu-miR-192-5p |
| D12 | mmu-miR-192-3p |
| D13 | mmu-miR-193a-3p |
| D14 | mmu-miR-193b-3p |
| D15 | mmu-miR-194-5p |
| D16 | mmu-miR-195a-5p |
| D17 | mmu-miR-196a-5p |
| D18 | mmu-miR-196b-5p |
| D19 | mmu-miR-196b-3p |
| D20 | mmu-miR-199a-3p |
| D21 | mmu-miR-199a-5p |
| D22 | mmu-miR-199b-5p |
| D23 | mmu-miR-19a-3p |
| D24 | mmu-miR-19b-3p |
| E01 | mmu-miR-1a-3p |
| E02 | mmu-miR-200a-3p |
| E03 | mmu-miR-200b-3p |
| E04 | mmu-miR-200c-3p |
| E05 | mmu-miR-203-3p |
| E06 | mmu-miR-204-5p |
| E07 | mmu-miR-204-3p |
| E08 | mmu-miR-205-5p |
| E09 | mmu-miR-206-3p |
| E10 | mmu-miR-206-5p |
| E11 | mmu-miR-207 |
| E12 | mmu-miR-20a-5p |
| E13 | mmu-miR-20b-5p |
| E14 | mmu-miR-21a-5p |
| E15 | mmu-miR-21a-3p |
| E16 | mmu-miR-210-3p |
| E17 | mmu-miR-211-5p |
| E18 | mmu-miR-214-3p |
| E19 | mmu-miR-215-5p |
| E20 | mmu-miR-216a-5p |
| E21 | mmu-miR-217-5p |
| E22 | mmu-miR-217-3p |
| E23 | mmu-miR-218-5p |
| E24 | mmu-miR-22-3p |
| F01 | mmu-miR-22-5p |
| F02 | mmu-miR-221-3p |
| F03 | mmu-miR-221-5p |
| F04 | mmu-miR-222-3p |
| F05 | mmu-miR-222-5p |
| F06 | mmu-miR-223-3p |
| F07 | mmu-miR-224-5p |
| F08 | mmu-miR-23a-3p |
| F09 | mmu-miR-23b-3p |
| F10 | mmu-miR-23b-5p |
| F11 | mmu-miR-24-3p |
| F12 | mmu-miR-25-3p |
| F13 | mmu-miR-26a-5p |
| F14 | mmu-miR-26b-5p |
| F15 | mmu-miR-27a-3p |
| F16 | mmu-miR-27b-3p |
| F17 | mmu-miR-27b-5p |
| F18 | mmu-miR-28a-5p |
| F19 | mmu-miR-292-3p |
| F20 | mmu-miR-295-3p |
| F21 | mmu-miR-298-5p |
| F22 | mmu-miR-29a-3p |
| F23 | mmu-miR-29b-3p |
| F24 | mmu-miR-29c-3p |
| G01 | mmu-miR-301a-3p |
| G02 | mmu-miR-302a-5p |
| G03 | mmu-miR-302d-3p |
| G04 | mmu-miR-30a-5p |
| G05 | mmu-miR-30b-5p |
| G06 | mmu-miR-30b-3p |
| G07 | mmu-miR-30c-5p |
| G08 | mmu-miR-30d-5p |
| G09 | mmu-miR-30e-5p |
| G10 | mmu-miR-31-5p |
| G11 | mmu-miR-31-3p |
| G12 | mmu-miR-32-5p |
| G13 | mmu-miR-320-3p |
| G14 | mmu-miR-322-5p |
| G15 | mmu-miR-324-5p |
| G16 | mmu-miR-326-3p |
| G17 | mmu-miR-328-3p |
| G18 | mmu-miR-33-5p |
| G19 | mmu-miR-330-5p |
| G20 | mmu-miR-335-5p |
| G21 | mmu-miR-338-3p |
| G22 | mmu-miR-339-5p |
| G23 | mmu-miR-342-3p |
| G24 | mmu-miR-345-5p |
| H01 | mmu-miR-346-5p |
| H02 | mmu-miR-34a-5p |
| H03 | mmu-miR-34c-5p |
| H04 | mmu-miR-34c-3p |
| H05 | mmu-miR-375-3p |
| H06 | mmu-miR-376b-3p |
| H07 | mmu-miR-376c-3p |
| H08 | mmu-miR-378a-3p |
| H09 | mmu-miR-378a-5p |
| H10 | mmu-miR-381-3p |
| H11 | mmu-miR-383-5p |
| H12 | mmu-miR-409-3p |
| H13 | mmu-miR-423-5p |
| H14 | mmu-miR-425-5p |
| H15 | mmu-miR-429-3p |
| H16 | mmu-miR-431-5p |
| H17 | mmu-miR-433-3p |
| H18 | mmu-miR-451a |
| H19 | mmu-miR-467e-5p |
| H20 | mmu-miR-484 |
| H21 | mmu-miR-485-5p |
| H22 | mmu-miR-493-3p |
| H23 | mmu-miR-495-3p |
| H24 | mmu-miR-497-5p |
| I01 | mmu-miR-499-5p |
| I02 | mmu-miR-503-5p |
| I03 | mmu-miR-503-3p |
| I04 | mmu-miR-541-5p |
| I05 | mmu-miR-574-3p |
| I06 | mmu-miR-598-3p |
| I07 | mmu-miR-652-3p |
| I08 | mmu-miR-744-5p |
| I09 | mmu-miR-7a-5p |
| I10 | mmu-miR-872-5p |
| I11 | mmu-miR-880-3p |
| I12 | mmu-miR-9-5p |
| I13 | mmu-miR-92a-3p |
| I14 | mmu-miR-93-5p |
| I15 | mmu-miR-96-5p |
| I16 | mmu-miR-98-5p |
| I17 | mmu-miR-99a-5p |
| I18 | mmu-miR-127-3p |
| I19 | mmu-miR-296-3p |
| I20 | mmu-miR-30c-1-3p |
| I21 | mmu-miR-34b-3p |
| I22 | mmu-miR-367-3p |
| I23 | mmu-miR-677-3p |
| I24 | mmu-miR-709 |
| J01 | mmu-miR-714 |
| J02 | mmu-miR-762 |
| J03 | mmu-miR-1b-5p |
| J04 | mmu-miR-212-3p |
| J05 | mmu-miR-219a-1-3p |
| J06 | mmu-miR-219a-5p |
| J07 | mmu-miR-290a-3p |
| J08 | mmu-miR-335-3p |
| J09 | mmu-miR-338-5p |
| J10 | mmu-miR-377-3p |
| J11 | mmu-miR-486-5p |
| J12 | mmu-miR-1187 |
| J13 | mmu-miR-1191 |
| J14 | mmu-miR-1192 |
| J15 | mmu-miR-1195 |
| J16 | mmu-miR-1196-5p |
| J17 | mmu-miR-1224-5p |
| J18 | mmu-miR-1249-3p |
| J19 | mmu-miR-1298-5p |
| J20 | mmu-miR-135a-1-3p |
| J21 | mmu-miR-136-5p |
| J22 | mmu-miR-154-5p |
| J23 | mmu-miR-16-1-3p |
| J24 | mmu-miR-188-3p |
| K01 | mmu-miR-188-5p |
| K02 | mmu-miR-1895 |
| K03 | mmu-miR-1900 |
| K04 | mmu-miR-1901 |
| K05 | mmu-miR-1902 |
| K06 | mmu-miR-1904 |
| K07 | mmu-miR-1931 |
| K08 | mmu-miR-1934-5p |
| K09 | mmu-miR-1936 |
| K10 | mmu-miR-1940 |
| K11 | mmu-miR-1945 |
| K12 | mmu-miR-1953 |
| K13 | mmu-miR-1957a |
| K14 | mmu-miR-1963 |
| K15 | mmu-miR-1965 |
| K16 | mmu-miR-1968-3p |
| K17 | mmu-miR-1971 |
| K18 | mmu-miR-1983 |
| K19 | mmu-miR-19b-1-5p |
| K20 | mmu-miR-1a-1-5p |
| K21 | mmu-miR-1a-2-5p |
| K22 | mmu-miR-202-3p |
| K23 | mmu-miR-208a-3p |
| K24 | mmu-miR-208a-5p |
| L01 | mmu-miR-208b-3p |
| L02 | mmu-miR-2137 |
| L03 | mmu-miR-2183 |
| L04 | mmu-miR-24-2-5p |
| L05 | mmu-miR-291b-3p |
| L06 | mmu-miR-293-5p |
| L07 | mmu-miR-294-3p |
| L08 | mmu-miR-302a-3p |
| L09 | mmu-miR-302b-3p |
| L10 | mmu-miR-3099-3p |
| L11 | mmu-miR-30c-2-3p |
| L12 | mmu-miR-331-3p |
| L13 | mmu-miR-341-3p |
| L14 | mmu-miR-343 |
| L15 | mmu-miR-344d-3-5p |
| L16 | mmu-miR-350-3p |
| L17 | mmu-miR-351-3p |
| L18 | mmu-miR-361-5p |
| L19 | mmu-miR-363-3p |
| L20 | mmu-miR-363-5p |
| L21 | mmu-miR-365-3p |
| L22 | mmu-miR-374b-5p |
| L23 | mmu-miR-376a-3p |
| L24 | mmu-miR-379-5p |
| M01 | mmu-miR-382-5p |
| M02 | mmu-miR-410-3p |
| M03 | mmu-miR-421-3p |
| M04 | mmu-miR-423-3p |
| M05 | mmu-miR-434-3p |
| M06 | mmu-miR-434-5p |
| M07 | mmu-miR-448-3p |
| M08 | mmu-miR-448-5p |
| M09 | mmu-miR-449a-5p |
| M10 | mmu-miR-449b |
| M11 | mmu-miR-449c-5p |
| M12 | mmu-miR-450a-5p |
| M13 | mmu-miR-452-3p |
| M14 | mmu-miR-452-5p |
| M15 | mmu-miR-455-3p |
| M16 | mmu-miR-466d-3p |
| M17 | mmu-miR-466h-5p |
| M18 | mmu-miR-468-3p |
| M19 | mmu-miR-470-5p |
| M20 | mmu-miR-483-5p |
| M21 | mmu-miR-483-3p |
| M22 | mmu-miR-490-3p |
| M23 | mmu-miR-490-5p |
| M24 | mmu-miR-491-5p |
| N01 | mmu-miR-496a-3p |
| N02 | mmu-miR-500-3p |
| N03 | mmu-miR-501-3p |
| N04 | mmu-miR-501-5p |
| N05 | mmu-miR-504-5p |
| N06 | mmu-miR-505-3p |
| N07 | mmu-miR-532-5p |
| N08 | mmu-miR-539-3p |
| N09 | mmu-miR-539-5p |
| N10 | mmu-miR-540-3p |
| N11 | mmu-miR-540-5p |
| N12 | mmu-miR-542-5p |
| N13 | mmu-miR-546 |
| N14 | mmu-miR-568 |
| N15 | mmu-miR-582-5p |
| N16 | mmu-miR-592-3p |
| N17 | mmu-miR-653-5p |
| N18 | mmu-miR-664-3p |
| N19 | mmu-miR-667-3p |
| N20 | mmu-miR-669a-5p |
| N21 | mmu-miR-669c-5p |
| N22 | mmu-miR-670-5p |
| N23 | mmu-miR-673-3p |
| N24 | mmu-miR-674-5p |
| O01 | mmu-miR-675-3p |
| O02 | mmu-miR-675-5p |
| O03 | mmu-miR-676-3p |
| O04 | mmu-miR-679-5p |
| O05 | mmu-miR-681 |
| O06 | mmu-miR-682 |
| O07 | mmu-miR-691 |
| O08 | mmu-miR-694 |
| O09 | mmu-miR-695 |
| O10 | mmu-miR-696 |
| O11 | mmu-miR-697 |
| O12 | mmu-miR-698-3p |
| O13 | mmu-miR-701-5p |
| O14 | mmu-miR-704 |
| O15 | mmu-miR-708-5p |
| O16 | mmu-miR-710 |
| O17 | mmu-miR-711 |
| O18 | mmu-miR-712-5p |
| O19 | mmu-miR-713 |
| O20 | mmu-miR-717 |
| O21 | mmu-miR-719 |
| O22 | mmu-miR-720 |
| O23 | mmu-miR-721 |
| O24 | mmu-miR-758-3p |
| P01 | mmu-miR-761 |
| P02 | mmu-miR-763 |
| P03 | mmu-miR-764-3p |
| P04 | mmu-miR-764-5p |
| P05 | mmu-miR-802-5p |
| P06 | mmu-miR-804 |
| P07 | mmu-miR-871-3p |
| P08 | mmu-miR-871-5p |
| P09 | mmu-miR-875-3p |
| P10 | mmu-miR-877-5p |
| P11 | mmu-miR-879-5p |
| P12 | mmu-miR-883b-5p |
| P13 | cel-miR-39-3p |
| P14 | cel-miR-39-3p |
| P15 | SNORD61 |
| P16 | SNORD68 |
| P17 | SNORD72 |
| P18 | SNORD95 |
| P19 | SNORD96A |
| P20 | RNU6-2 |
| P21 | miRTC |
| P22 | miRTC |
| P23 | PPC |
| P24 | PPC |

[Table B] Target gene analysis from the upregulated miRNAs. The list shows the overlapping target genes from TargetScan and miTarBase.

| **OFFICIAL_GENE_SYMBOL** | **Gene Name** |
| --- | --- |
| Adcy9 | [adenylate cyclase 9](http://david.abcc.ncifcrf.gov/geneReportFull.jsp?rowids=437470) |
| Arntl | [aryl hydrocarbon receptor nuclear translocator-like](http://david.abcc.ncifcrf.gov/geneReportFull.jsp?rowids=478116) |
| Bak1 | [BCL2-antagonist/killer 1](http://david.abcc.ncifcrf.gov/geneReportFull.jsp?rowids=444821) |
| Bbc3 | [BCL2 binding component 3](http://david.abcc.ncifcrf.gov/geneReportFull.jsp?rowids=424865) |
| Bmf | [BCL2 modifying factor](http://david.abcc.ncifcrf.gov/geneReportFull.jsp?rowids=426374) |
| CELF6 | [bruno-like 6, RNA binding protein (Drosophila)](http://david.abcc.ncifcrf.gov/geneReportFull.jsp?rowids=456196) |
| Calm1 | [predicted gene 7743; calmodulin 3; calmodulin 2; calmodulin 1; predicted gene 7308](http://david.abcc.ncifcrf.gov/geneReportFull.jsp?rowids=424317) |
| Calm2 | [predicted gene 7743; calmodulin 3; calmodulin 2; calmodulin 1; predicted gene 7308](http://david.abcc.ncifcrf.gov/geneReportFull.jsp?rowids=424317) |
| Casp9 | [caspase 9](http://david.abcc.ncifcrf.gov/geneReportFull.jsp?rowids=432202) |
| Cav2 | [caveolin 2](http://david.abcc.ncifcrf.gov/geneReportFull.jsp?rowids=442458) |
| Ccnd2 | [cyclin D2](http://david.abcc.ncifcrf.gov/geneReportFull.jsp?rowids=423915) |
| Cdc42 | [cell division cycle 42 homolog (S. cerevisiae); predicted gene 7407](http://david.abcc.ncifcrf.gov/geneReportFull.jsp?rowids=452546) |
| Cdk9 | [cyclin-dependent kinase 9 (CDC2-related kinase)](http://david.abcc.ncifcrf.gov/geneReportFull.jsp?rowids=424156) |
| Col1a1 | [collagen, type I, alpha 1](http://david.abcc.ncifcrf.gov/geneReportFull.jsp?rowids=441342) |
| Col1a2 | [collagen, type I, alpha 2](http://david.abcc.ncifcrf.gov/geneReportFull.jsp?rowids=476693) |
| Col2a1 | [collagen, type II, alpha 1](http://david.abcc.ncifcrf.gov/geneReportFull.jsp?rowids=431930) |
| Col3a1 | [collagen, type III, alpha 1](http://david.abcc.ncifcrf.gov/geneReportFull.jsp?rowids=451574) |
| Col4a2 | [collagen, type IV, alpha 2](http://david.abcc.ncifcrf.gov/geneReportFull.jsp?rowids=445082) |
| Col5a3 | [collagen, type V, alpha 3](http://david.abcc.ncifcrf.gov/geneReportFull.jsp?rowids=465980) |
| Ctnnbip1 | [catenin beta interacting protein 1](http://david.abcc.ncifcrf.gov/geneReportFull.jsp?rowids=457792) |
| Cygb | [cytoglobin](http://david.abcc.ncifcrf.gov/geneReportFull.jsp?rowids=468328) |
| Dnajc14 | [DnaJ (Hsp40) homolog, subfamily C, member 14](http://david.abcc.ncifcrf.gov/geneReportFull.jsp?rowids=468212) |
| Dnmt3a | [DNA methyltransferase 3A](http://david.abcc.ncifcrf.gov/geneReportFull.jsp?rowids=438006) |
| Dnmt3b | [DNA methyltransferase 3B](http://david.abcc.ncifcrf.gov/geneReportFull.jsp?rowids=464499) |
| Dusp2 | [dual specificity phosphatase 2](http://david.abcc.ncifcrf.gov/geneReportFull.jsp?rowids=449266) |
| Eln | [elastin](http://david.abcc.ncifcrf.gov/geneReportFull.jsp?rowids=471386) |
| Fbn1 | [fibrillin 1](http://david.abcc.ncifcrf.gov/geneReportFull.jsp?rowids=469019) |
| Fn1 | [fibronectin 1](http://david.abcc.ncifcrf.gov/geneReportFull.jsp?rowids=482407) |
| Fndc3b | [fibronectin type III domain containing 3B](http://david.abcc.ncifcrf.gov/geneReportFull.jsp?rowids=456688) |
| Foxp2 | [forkhead box P2](http://david.abcc.ncifcrf.gov/geneReportFull.jsp?rowids=467696) |
| Gja1 | [gap junction protein, alpha 1](http://david.abcc.ncifcrf.gov/geneReportFull.jsp?rowids=440260) |
| Hand2 | [heart and neural crest derivatives expressed transcript 2](http://david.abcc.ncifcrf.gov/geneReportFull.jsp?rowids=474561) |
| Hdac4 | [histone deacetylase 4](http://david.abcc.ncifcrf.gov/geneReportFull.jsp?rowids=458104) |
| Hiatl1 | [hippocampus abundant transcript-like 1; S100 calcium binding protein A4](http://david.abcc.ncifcrf.gov/geneReportFull.jsp?rowids=442554) |
| Hnrnpa2b1 | [predicted gene 5778; similar to heterogeneous nuclear ribonucleoprotein A2/B1; heterogeneous nuclear ribonucleoprotein A2/B1](http://david.abcc.ncifcrf.gov/geneReportFull.jsp?rowids=424828) |
| Hspd1 | [predicted gene 12141; heat shock protein 1 (chaperonin)](http://david.abcc.ncifcrf.gov/geneReportFull.jsp?rowids=464450) |
| Ifng | [interferon gamma](http://david.abcc.ncifcrf.gov/geneReportFull.jsp?rowids=429735) |
| Igf1 | [insulin-like growth factor 1](http://david.abcc.ncifcrf.gov/geneReportFull.jsp?rowids=444162) |
| Igf1r | [insulin-like growth factor I receptor](http://david.abcc.ncifcrf.gov/geneReportFull.jsp?rowids=477694) |
| Insig1 | [insulin induced gene 1](http://david.abcc.ncifcrf.gov/geneReportFull.jsp?rowids=431801) |
| Irak1 | [interleukin-1 receptor-associated kinase 1](http://david.abcc.ncifcrf.gov/geneReportFull.jsp?rowids=480835) |
| Irak2 | [interleukin-1 receptor-associated kinase 2](http://david.abcc.ncifcrf.gov/geneReportFull.jsp?rowids=453932) |
| Klf4 | [Kruppel-like factor 4 (gut)](http://david.abcc.ncifcrf.gov/geneReportFull.jsp?rowids=439346) |
| Med1 | [mediator complex subunit 1](http://david.abcc.ncifcrf.gov/geneReportFull.jsp?rowids=437034) |
| Mfap3 | [microfibrillar-associated protein 3](http://david.abcc.ncifcrf.gov/geneReportFull.jsp?rowids=451160) |
| Mtmr6 | [myotubularin related protein 6](http://david.abcc.ncifcrf.gov/geneReportFull.jsp?rowids=425912) |
| Nfatc4 | [nuclear factor of activated T-cells, cytoplasmic, calcineurin-dependent 4](http://david.abcc.ncifcrf.gov/geneReportFull.jsp?rowids=441919) |
| Notch1 | [Notch gene homolog 1 (Drosophila)](http://david.abcc.ncifcrf.gov/geneReportFull.jsp?rowids=453941) |
| Nrp1 | [neuropilin 1](http://david.abcc.ncifcrf.gov/geneReportFull.jsp?rowids=475651) |
| Papolg | [poly(A) polymerase gamma](http://david.abcc.ncifcrf.gov/geneReportFull.jsp?rowids=469072) |
| Pax7 | [paired box gene 7](http://david.abcc.ncifcrf.gov/geneReportFull.jsp?rowids=479159) |
| Phip | [pleckstrin homology domain interacting protein](http://david.abcc.ncifcrf.gov/geneReportFull.jsp?rowids=441440) |
| Pola1 | [polymerase (DNA directed), alpha 1](http://david.abcc.ncifcrf.gov/geneReportFull.jsp?rowids=473107) |
| Ptch1 | [patched homolog 1](http://david.abcc.ncifcrf.gov/geneReportFull.jsp?rowids=431478) |
| Rasa1 | [RAS p21 protein activator 1](http://david.abcc.ncifcrf.gov/geneReportFull.jsp?rowids=442033) |
| Rbak | [RB-associated KRAB repressor](http://david.abcc.ncifcrf.gov/geneReportFull.jsp?rowids=441549) |
| Rhoa | [ras homolog gene family, member A; similar to aplysia ras-related homolog A2; predicted gene 12844](http://david.abcc.ncifcrf.gov/geneReportFull.jsp?rowids=432091) |
| Rlim | [ring finger protein, LIM domain interacting](http://david.abcc.ncifcrf.gov/geneReportFull.jsp?rowids=451364) |
| Runx2 | [runt related transcription factor 2](http://david.abcc.ncifcrf.gov/geneReportFull.jsp?rowids=424891) |
| Spry1 | [sprouty homolog 1 (Drosophila); similar to sprouty 1](http://david.abcc.ncifcrf.gov/geneReportFull.jsp?rowids=425070) |
| Srf | [serum response factor](http://david.abcc.ncifcrf.gov/geneReportFull.jsp?rowids=439131) |
| Stat1 | [signal transducer and activator of transcription 1](http://david.abcc.ncifcrf.gov/geneReportFull.jsp?rowids=474327) |
| Tbc1d15 | [TBC1 domain family, member 15](http://david.abcc.ncifcrf.gov/geneReportFull.jsp?rowids=428409) |
| Tbx21 | [T-box 21](http://david.abcc.ncifcrf.gov/geneReportFull.jsp?rowids=476186) |
| Tcf4 | [transcription factor 4](http://david.abcc.ncifcrf.gov/geneReportFull.jsp?rowids=482380) |
| Tnrc6a | [trinucleotide repeat containing 6a](http://david.abcc.ncifcrf.gov/geneReportFull.jsp?rowids=470837) |
| Traf6 | [TNF receptor-associated factor 6](http://david.abcc.ncifcrf.gov/geneReportFull.jsp?rowids=480346) |
| Ucp2 | [uncoupling protein 2 (mitochondrial, proton carrier)](http://david.abcc.ncifcrf.gov/geneReportFull.jsp?rowids=463662) |
| Whsc2 | [Wolf-Hirschhorn syndrome candidate 2 (human)](http://david.abcc.ncifcrf.gov/geneReportFull.jsp?rowids=446789) |
| Yy1 | [YY1 transcription factor](http://david.abcc.ncifcrf.gov/geneReportFull.jsp?rowids=435509) |
| Zeb1 | [zinc finger E-box binding homeobox 1](http://david.abcc.ncifcrf.gov/geneReportFull.jsp?rowids=479390) |
| Zeb2 | [zinc finger E-box binding homeobox 2](http://david.abcc.ncifcrf.gov/geneReportFull.jsp?rowids=469200) |
| Srsf1 |  |

[Table C] Target gene analysis from the dwonregulated miRNAs. The list is the overlapping target genes from TargetScan and miTarBase.

| **OFFICIAL_GENE_SYMBOL** | **Gene Name** |
| --- | --- |
| Ahr | [aryl-hydrocarbon receptor](http://david.abcc.ncifcrf.gov/geneReportFull.jsp?rowids=473953) |
| Elavl4 | [ELAV (embryonic lethal, abnormal vision, Drosophila)-like 4 (Hu antigen D)](http://david.abcc.ncifcrf.gov/geneReportFull.jsp?rowids=458739) |
| Jak2 | [Janus kinase 2](http://david.abcc.ncifcrf.gov/geneReportFull.jsp?rowids=435649) |
| Mtpn | [myotrophin](http://david.abcc.ncifcrf.gov/geneReportFull.jsp?rowids=461005) |
| Usp1 | [ubiquitin specific peptidase 1; predicted gene 5841](http://david.abcc.ncifcrf.gov/geneReportFull.jsp?rowids=434041) |
| Yap1 | [yes-associated protein 1](http://david.abcc.ncifcrf.gov/geneReportFull.jsp?rowids=437901) |

[Table D] Functional analysis of the target genes performed by DAVID and KEGG

| **Gene Symbol** | **Gene Names** | **KEGG_PATHWAY** |
| --- | --- | --- |
| **Bbc3** | [**BCL2 binding component 3**](http://david.abcc.ncifcrf.gov/geneReportFull.jsp?rowids=424865) | [p53 signaling pathway](http://david.abcc.ncifcrf.gov/kegg.jsp?path=mmu04115$p53%20signaling%20pathway&termId=470049245&source=kegg), [Huntington's disease](http://david.abcc.ncifcrf.gov/kegg.jsp?path=mmu05016$Huntington's%20disease&termId=470049303&source=kegg), |
| **Dnmt3a** | [**DNA methyltransferase 3A**](http://david.abcc.ncifcrf.gov/geneReportFull.jsp?rowids=438006) | [Cysteine and methionine metabolism](http://david.abcc.ncifcrf.gov/kegg.jsp?path=mmu00270$Cysteine%20and%20methionine%20metabolism&termId=470049156&source=kegg), |
| **Dnmt3b** | [**DNA methyltransferase 3B**](http://david.abcc.ncifcrf.gov/geneReportFull.jsp?rowids=464499) | [Cysteine and methionine metabolism](http://david.abcc.ncifcrf.gov/kegg.jsp?path=mmu00270$Cysteine%20and%20methionine%20metabolism&termId=470049156&source=kegg) |
| **Notch1** | [**Notch gene homolog 1 (Drosophila)**](http://david.abcc.ncifcrf.gov/geneReportFull.jsp?rowids=453941) | [Dorso-ventral axis formation](http://david.abcc.ncifcrf.gov/kegg.jsp?path=mmu04320$Dorso-ventral%20axis%20formation&termId=470049256&source=kegg), [Notch signaling pathway](http://david.abcc.ncifcrf.gov/kegg.jsp?path=mmu04330$Notch%20signaling%20pathway&termId=470049257&source=kegg), [Prion diseases](http://david.abcc.ncifcrf.gov/kegg.jsp?path=mmu05020$Prion%20diseases&termId=470049304&source=kegg), |
| **Rasa1** | [**RAS p21 protein activator 1**](http://david.abcc.ncifcrf.gov/geneReportFull.jsp?rowids=442033) | [MAPK signaling pathway](http://david.abcc.ncifcrf.gov/kegg.jsp?path=mmu04010$MAPK%20signaling%20pathway&termId=470049236&source=kegg), [Axon guidance](http://david.abcc.ncifcrf.gov/kegg.jsp?path=mmu04360$Axon%20guidance&termId=470049260&source=kegg), |
| **Traf6** | [**TNF receptor-associated factor 6**](http://david.abcc.ncifcrf.gov/geneReportFull.jsp?rowids=480346) | [MAPK signaling pathway](http://david.abcc.ncifcrf.gov/kegg.jsp?path=mmu04010$MAPK%20signaling%20pathway&termId=470049236&source=kegg), [Ubiquitin mediated proteolysis](http://david.abcc.ncifcrf.gov/kegg.jsp?path=mmu04120$Ubiquitin%20mediated%20proteolysis&termId=470049246&source=kegg), [Endocytosis](http://david.abcc.ncifcrf.gov/kegg.jsp?path=mmu04144$Endocytosis&termId=470049250&source=kegg), [Toll-like receptor signaling pathway](http://david.abcc.ncifcrf.gov/kegg.jsp?path=mmu04620$Toll-like%20receptor%20signaling%20pathway&termId=470049271&source=kegg), [NOD-like receptor signaling pathway](http://david.abcc.ncifcrf.gov/kegg.jsp?path=mmu04621$NOD-like%20receptor%20signaling%20pathway&termId=470049272&source=kegg), [RIG-I-like receptor signaling pathway](http://david.abcc.ncifcrf.gov/kegg.jsp?path=mmu04622$RIG-I-like%20receptor%20signaling%20pathway&termId=470049273&source=kegg), [Neurotrophin signaling pathway](http://david.abcc.ncifcrf.gov/kegg.jsp?path=mmu04722$Neurotrophin%20signaling%20pathway&termId=470049286&source=kegg), [Pathways in cancer](http://david.abcc.ncifcrf.gov/kegg.jsp?path=mmu05200$Pathways%20in%20cancer&termId=470049305&source=kegg), [Small cell lung cancer](http://david.abcc.ncifcrf.gov/kegg.jsp?path=mmu05222$Small%20cell%20lung%20cancer&termId=470049318&source=kegg), |
| **Adcy9** | [**adenylate cyclase 9**](http://david.abcc.ncifcrf.gov/geneReportFull.jsp?rowids=437470) | [Purine metabolism](http://david.abcc.ncifcrf.gov/kegg.jsp?path=mmu00230$Purine%20metabolism&termId=470049151&source=kegg), [Calcium signaling pathway](http://david.abcc.ncifcrf.gov/kegg.jsp?path=mmu04020$Calcium%20signaling%20pathway&termId=470049238&source=kegg), [Chemokine signaling pathway](http://david.abcc.ncifcrf.gov/kegg.jsp?path=mmu04062$Chemokine%20signaling%20pathway&termId=470049240&source=kegg), [Oocyte meiosis](http://david.abcc.ncifcrf.gov/kegg.jsp?path=mmu04114$Oocyte%20meiosis&termId=470049244&source=kegg), [Vascular smooth muscle contraction](http://david.abcc.ncifcrf.gov/kegg.jsp?path=mmu04270$Vascular%20smooth%20muscle%20contraction&termId=470049254&source=kegg), [Gap junction](http://david.abcc.ncifcrf.gov/kegg.jsp?path=mmu04540$Gap%20junction&termId=470049267&source=kegg), [GnRH signaling pathway](http://david.abcc.ncifcrf.gov/kegg.jsp?path=mmu04912$GnRH%20signaling%20pathway&termId=470049292&source=kegg), [Progesterone-mediated oocyte maturation](http://david.abcc.ncifcrf.gov/kegg.jsp?path=mmu04914$Progesterone-mediated%20oocyte%20maturation&termId=470049293&source=kegg), [Melanogenesis](http://david.abcc.ncifcrf.gov/kegg.jsp?path=mmu04916$Melanogenesis&termId=470049294&source=kegg), [Dilated cardiomyopathy](http://david.abcc.ncifcrf.gov/kegg.jsp?path=mmu05414$Dilated%20cardiomyopathy&termId=470049328&source=kegg), |
| **Arntl** | [**aryl hydrocarbon receptor nuclear translocator-like**](http://david.abcc.ncifcrf.gov/geneReportFull.jsp?rowids=478116) | [Circadian rhythm](http://david.abcc.ncifcrf.gov/kegg.jsp?path=mmu04710$Circadian%20rhythm&termId=470049284&source=kegg) |
| **Casp9** | [**caspase 9**](http://david.abcc.ncifcrf.gov/geneReportFull.jsp?rowids=432202) | [p53 signaling pathway](http://david.abcc.ncifcrf.gov/kegg.jsp?path=mmu04115$p53%20signaling%20pathway&termId=470049245&source=kegg), [Apoptosis](http://david.abcc.ncifcrf.gov/kegg.jsp?path=mmu04210$Apoptosis&termId=470049252&source=kegg), [VEGF signaling pathway](http://david.abcc.ncifcrf.gov/kegg.jsp?path=mmu04370$VEGF%20signaling%20pathway&termId=470049261&source=kegg), [Alzheimer's disease](http://david.abcc.ncifcrf.gov/kegg.jsp?path=mmu05010$Alzheimer's%20disease&termId=470049300&source=kegg), [Parkinson's disease](http://david.abcc.ncifcrf.gov/kegg.jsp?path=mmu05012$Parkinson's%20disease&termId=470049301&source=kegg), [Amyotrophic lateral sclerosis (ALS)](http://david.abcc.ncifcrf.gov/kegg.jsp?path=mmu05014$Amyotrophic%20lateral%20sclerosis%20(ALS)&termId=470049302&source=kegg), [Huntington's disease](http://david.abcc.ncifcrf.gov/kegg.jsp?path=mmu05016$Huntington's%20disease&termId=470049303&source=kegg), [Pathways in cancer](http://david.abcc.ncifcrf.gov/kegg.jsp?path=mmu05200$Pathways%20in%20cancer&termId=470049305&source=kegg), [Colorectal cancer](http://david.abcc.ncifcrf.gov/kegg.jsp?path=mmu05210$Colorectal%20cancer&termId=470049306&source=kegg), [Pancreatic cancer](http://david.abcc.ncifcrf.gov/kegg.jsp?path=mmu05212$Pancreatic%20cancer&termId=470049308&source=kegg), [Endometrial cancer](http://david.abcc.ncifcrf.gov/kegg.jsp?path=mmu05213$Endometrial%20cancer&termId=470049309&source=kegg), [Prostate cancer](http://david.abcc.ncifcrf.gov/kegg.jsp?path=mmu05215$Prostate%20cancer&termId=470049311&source=kegg), [Small cell lung cancer](http://david.abcc.ncifcrf.gov/kegg.jsp?path=mmu05222$Small%20cell%20lung%20cancer&termId=470049318&source=kegg), [Non-small cell lung cancer](http://david.abcc.ncifcrf.gov/kegg.jsp?path=mmu05223$Non-small%20cell%20lung%20cancer&termId=470049319&source=kegg), [Viral myocarditis](http://david.abcc.ncifcrf.gov/kegg.jsp?path=mmu05416$Viral%20myocarditis&termId=470049329&source=kegg), |
| **Ctnnbip1** | [**catenin beta interacting protein 1**](http://david.abcc.ncifcrf.gov/geneReportFull.jsp?rowids=457792) | [Wnt signaling pathway](http://david.abcc.ncifcrf.gov/kegg.jsp?path=mmu04310$Wnt%20signaling%20pathway&termId=470049255&source=kegg) |
| **Cav2** | [**caveolin 2**](http://david.abcc.ncifcrf.gov/geneReportFull.jsp?rowids=442458) | [Focal adhesion](http://david.abcc.ncifcrf.gov/kegg.jsp?path=mmu04510$Focal%20adhesion&termId=470049262&source=kegg) |
| **Cdc42** | [**cell division cycle 42 homolog (S. cerevisiae); predicted gene 7407**](http://david.abcc.ncifcrf.gov/geneReportFull.jsp?rowids=452546) | [MAPK signaling pathway](http://david.abcc.ncifcrf.gov/kegg.jsp?path=mmu04010$MAPK%20signaling%20pathway&termId=470049236&source=kegg), [Chemokine signaling pathway](http://david.abcc.ncifcrf.gov/kegg.jsp?path=mmu04062$Chemokine%20signaling%20pathway&termId=470049240&source=kegg), [Endocytosis](http://david.abcc.ncifcrf.gov/kegg.jsp?path=mmu04144$Endocytosis&termId=470049250&source=kegg), [Axon guidance](http://david.abcc.ncifcrf.gov/kegg.jsp?path=mmu04360$Axon%20guidance&termId=470049260&source=kegg), [VEGF signaling pathway](http://david.abcc.ncifcrf.gov/kegg.jsp?path=mmu04370$VEGF%20signaling%20pathway&termId=470049261&source=kegg), [Focal adhesion](http://david.abcc.ncifcrf.gov/kegg.jsp?path=mmu04510$Focal%20adhesion&termId=470049262&source=kegg), [Adherens junction](http://david.abcc.ncifcrf.gov/kegg.jsp?path=mmu04520$Adherens%20junction&termId=470049265&source=kegg), [Tight junction](http://david.abcc.ncifcrf.gov/kegg.jsp?path=mmu04530$Tight%20junction&termId=470049266&source=kegg), [T cell receptor signaling pathway](http://david.abcc.ncifcrf.gov/kegg.jsp?path=mmu04660$T%20cell%20receptor%20signaling%20pathway&termId=470049278&source=kegg), [Fc gamma R-mediated phagocytosis](http://david.abcc.ncifcrf.gov/kegg.jsp?path=mmu04666$Fc%20gamma%20R-mediated%20phagocytosis&termId=470049281&source=kegg), [Leukocyte transendothelial migration](http://david.abcc.ncifcrf.gov/kegg.jsp?path=mmu04670$Leukocyte%20transendothelial%20migration&termId=470049282&source=kegg), [Neurotrophin signaling pathway](http://david.abcc.ncifcrf.gov/kegg.jsp?path=mmu04722$Neurotrophin%20signaling%20pathway&termId=470049286&source=kegg), [Regulation of actin cytoskeleton](http://david.abcc.ncifcrf.gov/kegg.jsp?path=mmu04810$Regulation%20of%20actin%20cytoskeleton&termId=470049290&source=kegg), [GnRH signaling pathway](http://david.abcc.ncifcrf.gov/kegg.jsp?path=mmu04912$GnRH%20signaling%20pathway&termId=470049292&source=kegg), [Pathways in cancer](http://david.abcc.ncifcrf.gov/kegg.jsp?path=mmu05200$Pathways%20in%20cancer&termId=470049305&source=kegg), [Renal cell carcinoma](http://david.abcc.ncifcrf.gov/kegg.jsp?path=mmu05211$Renal%20cell%20carcinoma&termId=470049307&source=kegg), [Pancreatic cancer](http://david.abcc.ncifcrf.gov/kegg.jsp?path=mmu05212$Pancreatic%20cancer&termId=470049308&source=kegg), |
| **Col1a1** | [**collagen, type I, alpha 1**](http://david.abcc.ncifcrf.gov/geneReportFull.jsp?rowids=441342) | [Focal adhesion](http://david.abcc.ncifcrf.gov/kegg.jsp?path=mmu04510$Focal%20adhesion&termId=470049262&source=kegg), [ECM-receptor interaction](http://david.abcc.ncifcrf.gov/kegg.jsp?path=mmu04512$ECM-receptor%20interaction&termId=470049263&source=kegg), |
| **Col1a2** | [**collagen, type I, alpha 2**](http://david.abcc.ncifcrf.gov/geneReportFull.jsp?rowids=476693) | [Focal adhesion](http://david.abcc.ncifcrf.gov/kegg.jsp?path=mmu04510$Focal%20adhesion&termId=470049262&source=kegg), [ECM-receptor interaction](http://david.abcc.ncifcrf.gov/kegg.jsp?path=mmu04512$ECM-receptor%20interaction&termId=470049263&source=kegg) |
| **Col2a1** | [**collagen, type II, alpha 1**](http://david.abcc.ncifcrf.gov/geneReportFull.jsp?rowids=431930) | [Focal adhesion](http://david.abcc.ncifcrf.gov/kegg.jsp?path=mmu04510$Focal%20adhesion&termId=470049262&source=kegg), [ECM-receptor interaction](http://david.abcc.ncifcrf.gov/kegg.jsp?path=mmu04512$ECM-receptor%20interaction&termId=470049263&source=kegg) |
| **Col3a1** | [**collagen, type III, alpha 1**](http://david.abcc.ncifcrf.gov/geneReportFull.jsp?rowids=451574) | [Focal adhesion](http://david.abcc.ncifcrf.gov/kegg.jsp?path=mmu04510$Focal%20adhesion&termId=470049262&source=kegg), [ECM-receptor interaction](http://david.abcc.ncifcrf.gov/kegg.jsp?path=mmu04512$ECM-receptor%20interaction&termId=470049263&source=kegg), |
| **Col4a2** | [**collagen, type IV, alpha 2**](http://david.abcc.ncifcrf.gov/geneReportFull.jsp?rowids=445082) | [Focal adhesion](http://david.abcc.ncifcrf.gov/kegg.jsp?path=mmu04510$Focal%20adhesion&termId=470049262&source=kegg), [ECM-receptor interaction](http://david.abcc.ncifcrf.gov/kegg.jsp?path=mmu04512$ECM-receptor%20interaction&termId=470049263&source=kegg), [Pathways in cancer](http://david.abcc.ncifcrf.gov/kegg.jsp?path=mmu05200$Pathways%20in%20cancer&termId=470049305&source=kegg), [Small cell lung cancer](http://david.abcc.ncifcrf.gov/kegg.jsp?path=mmu05222$Small%20cell%20lung%20cancer&termId=470049318&source=kegg), |
| **Col5a3** | [**collagen, type V, alpha 3**](http://david.abcc.ncifcrf.gov/geneReportFull.jsp?rowids=465980) | [Focal adhesion](http://david.abcc.ncifcrf.gov/kegg.jsp?path=mmu04510$Focal%20adhesion&termId=470049262&source=kegg), [ECM-receptor interaction](http://david.abcc.ncifcrf.gov/kegg.jsp?path=mmu04512$ECM-receptor%20interaction&termId=470049263&source=kegg), |
| **Ccnd2** | [**cyclin D2**](http://david.abcc.ncifcrf.gov/geneReportFull.jsp?rowids=423915) | [Cell cycle](http://david.abcc.ncifcrf.gov/kegg.jsp?path=mmu04110$Cell%20cycle&termId=470049243&source=kegg), [p53 signaling pathway](http://david.abcc.ncifcrf.gov/kegg.jsp?path=mmu04115$p53%20signaling%20pathway&termId=470049245&source=kegg), [Wnt signaling pathway](http://david.abcc.ncifcrf.gov/kegg.jsp?path=mmu04310$Wnt%20signaling%20pathway&termId=470049255&source=kegg), [Focal adhesion](http://david.abcc.ncifcrf.gov/kegg.jsp?path=mmu04510$Focal%20adhesion&termId=470049262&source=kegg), [Jak-STAT signaling pathway](http://david.abcc.ncifcrf.gov/kegg.jsp?path=mmu04630$Jak-STAT%20signaling%20pathway&termId=470049275&source=kegg), |
| **Dusp2** | [**dual specificity phosphatase 2**](http://david.abcc.ncifcrf.gov/geneReportFull.jsp?rowids=449266) | [MAPK signaling pathway](http://david.abcc.ncifcrf.gov/kegg.jsp?path=mmu04010$MAPK%20signaling%20pathway&termId=470049236&source=kegg), |
| **Fn1** | [**fibronectin 1**](http://david.abcc.ncifcrf.gov/geneReportFull.jsp?rowids=482407) | [Focal adhesion](http://david.abcc.ncifcrf.gov/kegg.jsp?path=mmu04510$Focal%20adhesion&termId=470049262&source=kegg), [ECM-receptor interaction](http://david.abcc.ncifcrf.gov/kegg.jsp?path=mmu04512$ECM-receptor%20interaction&termId=470049263&source=kegg), [Regulation of actin cytoskeleton](http://david.abcc.ncifcrf.gov/kegg.jsp?path=mmu04810$Regulation%20of%20actin%20cytoskeleton&termId=470049290&source=kegg), [Pathways in cancer](http://david.abcc.ncifcrf.gov/kegg.jsp?path=mmu05200$Pathways%20in%20cancer&termId=470049305&source=kegg), [Small cell lung cancer](http://david.abcc.ncifcrf.gov/kegg.jsp?path=mmu05222$Small%20cell%20lung%20cancer&termId=470049318&source=kegg), |
| **Gja1** | [**gap junction protein, alpha 1**](http://david.abcc.ncifcrf.gov/geneReportFull.jsp?rowids=440260) | [Gap junction](http://david.abcc.ncifcrf.gov/kegg.jsp?path=mmu04540$Gap%20junction&termId=470049267&source=kegg), [Arrhythmogenic right ventricular cardiomyopathy (ARVC)](http://david.abcc.ncifcrf.gov/kegg.jsp?path=mmu05412$Arrhythmogenic%20right%20ventricular%20cardiomyopathy%20(ARVC)&termId=470049327&source=kegg) |
| **Igf1** | [**insulin-like growth factor 1**](http://david.abcc.ncifcrf.gov/geneReportFull.jsp?rowids=444162) | [Oocyte meiosis](http://david.abcc.ncifcrf.gov/kegg.jsp?path=mmu04114$Oocyte%20meiosis&termId=470049244&source=kegg), [p53 signaling pathway](http://david.abcc.ncifcrf.gov/kegg.jsp?path=mmu04115$p53%20signaling%20pathway&termId=470049245&source=kegg), [mTOR signaling pathway](http://david.abcc.ncifcrf.gov/kegg.jsp?path=mmu04150$mTOR%20signaling%20pathway&termId=470049251&source=kegg), [Focal adhesion](http://david.abcc.ncifcrf.gov/kegg.jsp?path=mmu04510$Focal%20adhesion&termId=470049262&source=kegg), [Long-term depression](http://david.abcc.ncifcrf.gov/kegg.jsp?path=mmu04730$Long-term%20depression&termId=470049287&source=kegg), [Progesterone-mediated oocyte maturation](http://david.abcc.ncifcrf.gov/kegg.jsp?path=mmu04914$Progesterone-mediated%20oocyte%20maturation&termId=470049293&source=kegg), [Aldosterone-regulated sodium reabsorption](http://david.abcc.ncifcrf.gov/kegg.jsp?path=mmu04960$Aldosterone-regulated%20sodium%20reabsorption&termId=470049299&source=kegg), [Pathways in cancer](http://david.abcc.ncifcrf.gov/kegg.jsp?path=mmu05200$Pathways%20in%20cancer&termId=470049305&source=kegg), [Glioma](http://david.abcc.ncifcrf.gov/kegg.jsp?path=mmu05214$Glioma&termId=470049310&source=kegg), [Prostate cancer](http://david.abcc.ncifcrf.gov/kegg.jsp?path=mmu05215$Prostate%20cancer&termId=470049311&source=kegg), [Melanoma](http://david.abcc.ncifcrf.gov/kegg.jsp?path=mmu05218$Melanoma&termId=470049314&source=kegg), [Hypertrophic cardiomyopathy (HCM)](http://david.abcc.ncifcrf.gov/kegg.jsp?path=mmu05410$Hypertrophic%20cardiomyopathy%20(HCM)&termId=470049326&source=kegg), [Dilated cardiomyopathy](http://david.abcc.ncifcrf.gov/kegg.jsp?path=mmu05414$Dilated%20cardiomyopathy&termId=470049328&source=kegg), |
| **Igf1r** | [**insulin-like growth factor I receptor**](http://david.abcc.ncifcrf.gov/geneReportFull.jsp?rowids=477694) | [Oocyte meiosis](http://david.abcc.ncifcrf.gov/kegg.jsp?path=mmu04114$Oocyte%20meiosis&termId=470049244&source=kegg), [Endocytosis](http://david.abcc.ncifcrf.gov/kegg.jsp?path=mmu04144$Endocytosis&termId=470049250&source=kegg), [Focal adhesion](http://david.abcc.ncifcrf.gov/kegg.jsp?path=mmu04510$Focal%20adhesion&termId=470049262&source=kegg), [Adherens junction](http://david.abcc.ncifcrf.gov/kegg.jsp?path=mmu04520$Adherens%20junction&termId=470049265&source=kegg), [Long-term depression](http://david.abcc.ncifcrf.gov/kegg.jsp?path=mmu04730$Long-term%20depression&termId=470049287&source=kegg), [Progesterone-mediated oocyte maturation](http://david.abcc.ncifcrf.gov/kegg.jsp?path=mmu04914$Progesterone-mediated%20oocyte%20maturation&termId=470049293&source=kegg), [Pathways in cancer](http://david.abcc.ncifcrf.gov/kegg.jsp?path=mmu05200$Pathways%20in%20cancer&termId=470049305&source=kegg), [Colorectal cancer](http://david.abcc.ncifcrf.gov/kegg.jsp?path=mmu05210$Colorectal%20cancer&termId=470049306&source=kegg), [Glioma](http://david.abcc.ncifcrf.gov/kegg.jsp?path=mmu05214$Glioma&termId=470049310&source=kegg), [Prostate cancer](http://david.abcc.ncifcrf.gov/kegg.jsp?path=mmu05215$Prostate%20cancer&termId=470049311&source=kegg), [Melanoma](http://david.abcc.ncifcrf.gov/kegg.jsp?path=mmu05218$Melanoma&termId=470049314&source=kegg), |
| **Ifng** | [**interferon gamma**](http://david.abcc.ncifcrf.gov/geneReportFull.jsp?rowids=429735) | [Proteasome](http://david.abcc.ncifcrf.gov/kegg.jsp?path=mmu03050$Proteasome&termId=470049228&source=kegg), [Cytokine-cytokine receptor interaction](http://david.abcc.ncifcrf.gov/kegg.jsp?path=mmu04060$Cytokine-cytokine%20receptor%20interaction&termId=470049239&source=kegg), [Regulation of autophagy](http://david.abcc.ncifcrf.gov/kegg.jsp?path=mmu04140$Regulation%20of%20autophagy&termId=470049248&source=kegg), [TGF-beta signaling pathway](http://david.abcc.ncifcrf.gov/kegg.jsp?path=mmu04350$TGF-beta%20signaling%20pathway&termId=470049259&source=kegg), [Jak-STAT signaling pathway](http://david.abcc.ncifcrf.gov/kegg.jsp?path=mmu04630$Jak-STAT%20signaling%20pathway&termId=470049275&source=kegg), [Natural killer cell mediated cytotoxicity](http://david.abcc.ncifcrf.gov/kegg.jsp?path=mmu04650$Natural%20killer%20cell%20mediated%20cytotoxicity&termId=470049277&source=kegg), [T cell receptor signaling pathway](http://david.abcc.ncifcrf.gov/kegg.jsp?path=mmu04660$T%20cell%20receptor%20signaling%20pathway&termId=470049278&source=kegg), [Type I diabetes mellitus](http://david.abcc.ncifcrf.gov/kegg.jsp?path=mmu04940$Type%20I%20diabetes%20mellitus&termId=470049297&source=kegg), [Systemic lupus erythematosus](http://david.abcc.ncifcrf.gov/kegg.jsp?path=mmu05322$Systemic%20lupus%20erythematosus&termId=470049322&source=kegg), [Allograft rejection](http://david.abcc.ncifcrf.gov/kegg.jsp?path=mmu05330$Allograft%20rejection&termId=470049323&source=kegg), [Graft-versus-host disease](http://david.abcc.ncifcrf.gov/kegg.jsp?path=mmu05332$Graft-versus-host%20disease&termId=470049324&source=kegg), |
| **Irak1** | [**interleukin-1 receptor-associated kinase 1**](http://david.abcc.ncifcrf.gov/geneReportFull.jsp?rowids=480835) | [Apoptosis](http://david.abcc.ncifcrf.gov/kegg.jsp?path=mmu04210$Apoptosis&termId=470049252&source=kegg), [Toll-like receptor signaling pathway](http://david.abcc.ncifcrf.gov/kegg.jsp?path=mmu04620$Toll-like%20receptor%20signaling%20pathway&termId=470049271&source=kegg), [Neurotrophin signaling pathway](http://david.abcc.ncifcrf.gov/kegg.jsp?path=mmu04722$Neurotrophin%20signaling%20pathway&termId=470049286&source=kegg), |
| **Irak2** | [**interleukin-1 receptor-associated kinase 2**](http://david.abcc.ncifcrf.gov/geneReportFull.jsp?rowids=453932) | [Apoptosis](http://david.abcc.ncifcrf.gov/kegg.jsp?path=mmu04210$Apoptosis&termId=470049252&source=kegg), [Neurotrophin signaling pathway](http://david.abcc.ncifcrf.gov/kegg.jsp?path=mmu04722$Neurotrophin%20signaling%20pathway&termId=470049286&source=kegg), |
| **Mtmr6** | [**myotubularin related protein 6**](http://david.abcc.ncifcrf.gov/geneReportFull.jsp?rowids=425912) | [Fructose and mannose metabolism](http://david.abcc.ncifcrf.gov/kegg.jsp?path=mmu00051$Fructose%20and%20mannose%20metabolism&termId=470049138&source=kegg), [Thiamine metabolism](http://david.abcc.ncifcrf.gov/kegg.jsp?path=mmu00730$Thiamine%20metabolism&termId=470049202&source=kegg), [Riboflavin metabolism](http://david.abcc.ncifcrf.gov/kegg.jsp?path=mmu00740$Riboflavin%20metabolism&termId=470049203&source=kegg), |
| **Nrp1** | [**neuropilin 1**](http://david.abcc.ncifcrf.gov/geneReportFull.jsp?rowids=475651) | [Axon guidance](http://david.abcc.ncifcrf.gov/kegg.jsp?path=mmu04360$Axon%20guidance&termId=470049260&source=kegg), |
| **Nfatc4** | [**nuclear factor of activated T-cells, cytoplasmic, calcineurin-dependent 4**](http://david.abcc.ncifcrf.gov/geneReportFull.jsp?rowids=441919) | [MAPK signaling pathway](http://david.abcc.ncifcrf.gov/kegg.jsp?path=mmu04010$MAPK%20signaling%20pathway&termId=470049236&source=kegg), [Wnt signaling pathway](http://david.abcc.ncifcrf.gov/kegg.jsp?path=mmu04310$Wnt%20signaling%20pathway&termId=470049255&source=kegg), [Axon guidance](http://david.abcc.ncifcrf.gov/kegg.jsp?path=mmu04360$Axon%20guidance&termId=470049260&source=kegg), [VEGF signaling pathway](http://david.abcc.ncifcrf.gov/kegg.jsp?path=mmu04370$VEGF%20signaling%20pathway&termId=470049261&source=kegg), [Natural killer cell mediated cytotoxicity](http://david.abcc.ncifcrf.gov/kegg.jsp?path=mmu04650$Natural%20killer%20cell%20mediated%20cytotoxicity&termId=470049277&source=kegg), [T cell receptor signaling pathway](http://david.abcc.ncifcrf.gov/kegg.jsp?path=mmu04660$T%20cell%20receptor%20signaling%20pathway&termId=470049278&source=kegg), [B cell receptor signaling pathway](http://david.abcc.ncifcrf.gov/kegg.jsp?path=mmu04662$B%20cell%20receptor%20signaling%20pathway&termId=470049279&source=kegg), |
| **Ptch1** | [**patched homolog 1**](http://david.abcc.ncifcrf.gov/geneReportFull.jsp?rowids=431478) | [Hedgehog signaling pathway](http://david.abcc.ncifcrf.gov/kegg.jsp?path=mmu04340$Hedgehog%20signaling%20pathway&termId=470049258&source=kegg), [Pathways in cancer](http://david.abcc.ncifcrf.gov/kegg.jsp?path=mmu05200$Pathways%20in%20cancer&termId=470049305&source=kegg), [Basal cell carcinoma](http://david.abcc.ncifcrf.gov/kegg.jsp?path=mmu05217$Basal%20cell%20carcinoma&termId=470049313&source=kegg), |
| **Papolg** | [**poly(A) polymerase gamma**](http://david.abcc.ncifcrf.gov/geneReportFull.jsp?rowids=469072) | [RNA degradation](http://david.abcc.ncifcrf.gov/kegg.jsp?path=mmu03018$RNA%20degradation&termId=470049223&source=kegg), |
| **Pola1** | [**polymerase (DNA directed), alpha 1**](http://david.abcc.ncifcrf.gov/geneReportFull.jsp?rowids=473107) | [Purine metabolism](http://david.abcc.ncifcrf.gov/kegg.jsp?path=mmu00230$Purine%20metabolism&termId=470049151&source=kegg), [Pyrimidine metabolism](http://david.abcc.ncifcrf.gov/kegg.jsp?path=mmu00240$Pyrimidine%20metabolism&termId=470049153&source=kegg), [DNA replication](http://david.abcc.ncifcrf.gov/kegg.jsp?path=mmu03030$DNA%20replication&termId=470049226&source=kegg), |
| **Hspd1** | [**predicted gene 12141; heat shock protein 1 (chaperonin)**](http://david.abcc.ncifcrf.gov/geneReportFull.jsp?rowids=464450) | [RNA degradation](http://david.abcc.ncifcrf.gov/kegg.jsp?path=mmu03018$RNA%20degradation&termId=470049223&source=kegg), [Type I diabetes mellitus](http://david.abcc.ncifcrf.gov/kegg.jsp?path=mmu04940$Type%20I%20diabetes%20mellitus&termId=470049297&source=kegg), |
| **Calm1, Calm2** | [**predicted gene 7743; calmodulin 3; calmodulin 2; calmodulin 1; predicted gene 7308**](http://david.abcc.ncifcrf.gov/geneReportFull.jsp?rowids=424317) | [Calcium signaling pathway](http://david.abcc.ncifcrf.gov/kegg.jsp?path=mmu04020$Calcium%20signaling%20pathway&termId=470049238&source=kegg), [Phosphatidylinositol signaling system](http://david.abcc.ncifcrf.gov/kegg.jsp?path=mmu04070$Phosphatidylinositol%20signaling%20system&termId=470049241&source=kegg), [Oocyte meiosis](http://david.abcc.ncifcrf.gov/kegg.jsp?path=mmu04114$Oocyte%20meiosis&termId=470049244&source=kegg), [Vascular smooth muscle contraction](http://david.abcc.ncifcrf.gov/kegg.jsp?path=mmu04270$Vascular%20smooth%20muscle%20contraction&termId=470049254&source=kegg), [Long-term potentiation](http://david.abcc.ncifcrf.gov/kegg.jsp?path=mmu04720$Long-term%20potentiation&termId=470049285&source=kegg), [Neurotrophin signaling pathway](http://david.abcc.ncifcrf.gov/kegg.jsp?path=mmu04722$Neurotrophin%20signaling%20pathway&termId=470049286&source=kegg), [Olfactory transduction](http://david.abcc.ncifcrf.gov/kegg.jsp?path=mmu04740$Olfactory%20transduction&termId=470049288&source=kegg), [Insulin signaling pathway](http://david.abcc.ncifcrf.gov/kegg.jsp?path=mmu04910$Insulin%20signaling%20pathway&termId=470049291&source=kegg), [GnRH signaling pathway](http://david.abcc.ncifcrf.gov/kegg.jsp?path=mmu04912$GnRH%20signaling%20pathway&termId=470049292&source=kegg), [Melanogenesis](http://david.abcc.ncifcrf.gov/kegg.jsp?path=mmu04916$Melanogenesis&termId=470049294&source=kegg), [Alzheimer's disease](http://david.abcc.ncifcrf.gov/kegg.jsp?path=mmu05010$Alzheimer's%20disease&termId=470049300&source=kegg), [Glioma](http://david.abcc.ncifcrf.gov/kegg.jsp?path=mmu05214$Glioma&termId=470049310&source=kegg), |
| **Rhoa** | [**ras homolog gene family, member A; similar to aplysia ras-related homolog A2; predicted gene 12844**](http://david.abcc.ncifcrf.gov/geneReportFull.jsp?rowids=432091) | [Chemokine signaling pathway](http://david.abcc.ncifcrf.gov/kegg.jsp?path=mmu04062$Chemokine%20signaling%20pathway&termId=470049240&source=kegg), [Vascular smooth muscle contraction](http://david.abcc.ncifcrf.gov/kegg.jsp?path=mmu04270$Vascular%20smooth%20muscle%20contraction&termId=470049254&source=kegg), [Wnt signaling pathway](http://david.abcc.ncifcrf.gov/kegg.jsp?path=mmu04310$Wnt%20signaling%20pathway&termId=470049255&source=kegg), [TGF-beta signaling pathway](http://david.abcc.ncifcrf.gov/kegg.jsp?path=mmu04350$TGF-beta%20signaling%20pathway&termId=470049259&source=kegg), [Axon guidance](http://david.abcc.ncifcrf.gov/kegg.jsp?path=mmu04360$Axon%20guidance&termId=470049260&source=kegg), [Focal adhesion](http://david.abcc.ncifcrf.gov/kegg.jsp?path=mmu04510$Focal%20adhesion&termId=470049262&source=kegg), [Adherens junction](http://david.abcc.ncifcrf.gov/kegg.jsp?path=mmu04520$Adherens%20junction&termId=470049265&source=kegg), [Tight junction](http://david.abcc.ncifcrf.gov/kegg.jsp?path=mmu04530$Tight%20junction&termId=470049266&source=kegg), [T cell receptor signaling pathway](http://david.abcc.ncifcrf.gov/kegg.jsp?path=mmu04660$T%20cell%20receptor%20signaling%20pathway&termId=470049278&source=kegg), [Leukocyte transendothelial migration](http://david.abcc.ncifcrf.gov/kegg.jsp?path=mmu04670$Leukocyte%20transendothelial%20migration&termId=470049282&source=kegg), [Neurotrophin signaling pathway](http://david.abcc.ncifcrf.gov/kegg.jsp?path=mmu04722$Neurotrophin%20signaling%20pathway&termId=470049286&source=kegg), [Regulation of actin cytoskeleton](http://david.abcc.ncifcrf.gov/kegg.jsp?path=mmu04810$Regulation%20of%20actin%20cytoskeleton&termId=470049290&source=kegg), [Pathways in cancer](http://david.abcc.ncifcrf.gov/kegg.jsp?path=mmu05200$Pathways%20in%20cancer&termId=470049305&source=kegg), |
| **Srf** | [**serum response factor**](http://david.abcc.ncifcrf.gov/geneReportFull.jsp?rowids=439131) | [MAPK signaling pathway](http://david.abcc.ncifcrf.gov/kegg.jsp?path=mmu04010$MAPK%20signaling%20pathway&termId=470049236&source=kegg), |
| **Stat1** | [**signal transducer and activator of transcription 1**](http://david.abcc.ncifcrf.gov/geneReportFull.jsp?rowids=474327) | [Chemokine signaling pathway](http://david.abcc.ncifcrf.gov/kegg.jsp?path=mmu04062$Chemokine%20signaling%20pathway&termId=470049240&source=kegg), [Toll-like receptor signaling pathway](http://david.abcc.ncifcrf.gov/kegg.jsp?path=mmu04620$Toll-like%20receptor%20signaling%20pathway&termId=470049271&source=kegg), [Jak-STAT signaling pathway](http://david.abcc.ncifcrf.gov/kegg.jsp?path=mmu04630$Jak-STAT%20signaling%20pathway&termId=470049275&source=kegg), [Pathways in cancer](http://david.abcc.ncifcrf.gov/kegg.jsp?path=mmu05200$Pathways%20in%20cancer&termId=470049305&source=kegg), [Pancreatic cancer](http://david.abcc.ncifcrf.gov/kegg.jsp?path=mmu05212$Pancreatic%20cancer&termId=470049308&source=kegg), |
| **Spry1** | [**sprouty homolog 1 (Drosophila); similar to sprouty 1**](http://david.abcc.ncifcrf.gov/geneReportFull.jsp?rowids=425070) | [Jak-STAT signaling pathway](http://david.abcc.ncifcrf.gov/kegg.jsp?path=mmu04630$Jak-STAT%20signaling%20pathway&termId=470049275&source=kegg), |
